# Insights on the drivers of genetic divergence in the European anchovy

**DOI:** 10.1038/s41598-017-03926-z

**Published:** 2017-06-23

**Authors:** Gaetano Catanese, Romain Watteaux, Iratxe Montes, Marco Barra, Paola Rumolo, Diego Borme, Bruno Buongiorno Nardelli, Vincenzo Botte, Maria Grazia Mazzocchi, Simona Genovese, Iole Di Capua, Mikel Iriondo, Andone Estonba, Paolo Ruggeri, Valentina Tirelli, Vincenzo Caputo-Barucchi, Gualtiero Basilone, Angelo Bonanno, Daniele Iudicone, Gabriele Procaccini

**Affiliations:** 1 Department of Integrative Marine Ecology, Stazione Zoologica Anton Dohrn, Villa Comunale, 80121 Napoli, Italy; 2 Department of Genetics, Physical Anthropology and Animal Physiology, University of the Basque Country (UPV/EHU), Sarriena auzoa z/g, Leioa - Bizkaia, Spain; 3 Institute for Coastal and Marine Environment (IAMC), Consiglio Nazionale delle Ricerche (CNR), Detached Units of Capo Granitola (TP) and Naples, Napoli, Italy; 4 Department of Oceanography, Istituto Nazionale di Oceanografia e di Geofisica Sperimentale (OGS), Via A. Piccard 54, 34151 Trieste, Italy; 5 Dipartimento di Scienze della Vita e dell’Ambiente (DiSVA), Università Politecnica delle Marche, via Brecce Bianche, 60131 Ancona, Italy; 6 University of Miami, RSMAS, 4600 Rickenbacker Causeway, 33149 Miami, Florida USA; 7 Laboratorio de Investigaciones Marinas y Acuicultura (LIMIA) Govern de les Illes Balears, Av. Gabriel Roca 69, 07158 Port d’Andratx - Mallorca, Spain

## Abstract

Anchovies represent the largest world’s marine fish catches and the current threats on their populations impose a sustainable exploitment based on sound scientific information. In the European anchovy (*Engraulis encrasicolus*), the existence of several populations has been proposed but a global view is missing. Using a multidisciplinary approach, here we assessed the divergence among different ecotypes and its possible causes. SNPs have revealed two functionally distinct ecotypes overlapping in the Central Mediterranean, with one ecotype confined near the river estuaries. The same SNPs outliers also segregated two distinct populations in the near Atlantic, despite their large spatial distance. In addition, while most studies suggested that adaptation to low salinity is key to divergence, here we show that the offshore ecotype has higher environmental tolerance and an opportunistic feeding behaviour, as assessed by the study of environmental conditions, anchovy diet and trophic levels, and passive egg dispersal. These results provide insights into the anchovy evolutionary history, stressing the importance of behaviour in shaping ecotypes.

## Introduction

The European anchovy (*Engraulis encrasicolus*) represents one of the most important European fishery resources^1^. This species is ubiquitous from tropical to temperate areas of Atlantic Ocean, Mediterranean Sea, and Black Sea. Active dispersal of adult individuals plays an important role in species distribution. Moreover, the recruitment of European anchovy is in part influenced by pelagic transport of eggs and larvae from spawning to nursery areas and by diel vertical migration of larvae^2^.

European anchovy shows high levels of genetic structure attributed to habitat heterogeneity^3^, specific environmental features^4, 5^ and limited gene flow over wide geographic distances^6^. Overall, the European anchovy populations are thought to belong to two co-existing ecological groups (or ecotypes), named wide- (or marine) and narrow-shelf (or coastal), whose differences reflect ancient dispersal and colonization events of the species^6–9^. The current distribution of the marine ecotype is likely attributable to founding events into the Mediterranean Sea, Bay of Biscay and northern European seas by a common ancestral population that fled to the Mediterranean during last glaciation^6, 10^. In contrast, the current distribution of the coastal ecotype could derive from the colonization of the Atlantic front after the end of the last glaciation^6^ by an ancestral population probably sheltered in the West African coast^10^.

The estuarine/lagunar populations that have been morphometrically and genetically identified in the Mediterranean Sea, Bay of Biscay and North Sea^3, 9, 11–20^ could represent specific types of the coastal ecotype. These coastal populations are likely in the process of becoming reproductively isolated from other anchovy populations coexisting together in these basins^17, 20, 21^as result of parallel genetic differentiation prior to secondary contact^9, 17^ and to local adaptation^19, 20^.

In this study, we assessed the existence of two genetic/ecological ecotypes of *E. encrasicolus* in the Mediterranean Sea, namely coastal and marine (from hereafter called offshore), by characterizing anchovy populations from the Italian seas (Tyrrhenian, Ionian and Adriatic Seas) for a set of 96 gene-associated SNPs^22^. Additionally, we assessed the evolutionary divergence among the two ecotypes and its possible causes by applying a multi-faceted approach. We simulated current-driven dispersal of eggs and adults and related the presence of the two ecotypes to environmental factors, including zooplankton community, and trophic features. The genetic relationship between the two ecotype pairs in Mediterranean and in the Atlantic Sea was also analysed.

We found that: (i) in the Italian seas, populations of offshore and coastal anchovy ecotypes coexist, occupying partially overlapping niches; (ii) the SNP markers identified as putative outliers in the Mediterranean are among the outlier markers identified in the Bay of Biscay, suggesting allopatric convergent adaptation of the two ecotypes in the two geographic areas,; (iii) a strong differentiation exists also when considering only putatively neutral *loci*, suggesting high complexity in the evolutionary origin of the two ecotypes. According to fine-scale multidisciplinary analysis, we conclude that the offshore ecotype has higher environmental tolerance and opportunistic feeding behaviour and we stress the importance of behaviour in shaping population and ecotype boundaries.

## Results

### Population genetics

The number of adult anchovies and eggs successfully genotyped in each sampling site, for the 96 high-resolution SNP panel utilized, is reported in Table 1.

**Table 1:** Sampling informations: locations, number of anchovy (adults and egg) analysed (N), latitude, longitude, sampling date, percentage of genotypes coastal (C), offshore (O) and putative hybrids (h) for each sampling site. GSA: Geographical Sub-Areas of the General Fisheries Commission for the Mediterranean (GFCM).

	ID	N	LATITUDE	LONGITUDE	DATE	% C/O/h
**ADULTS**						
**South and Central Tyrrhenian (GSA 10)**						
Castellammare del Golfo*	GCM	30	38°06′N	13°01′E	May-13	0/70/30
Termini Imerese*	TRM	24	38°02′N	13°36′E	May-13	4/88/13
Paola	PAO	30	39°16′N	16°01′E	Jun-13	7/63/30
Diamante*	DIA	30	39°36′N	15°46′E	Jun-13	7/67/27
Capaccio*	CAP	30	40°27′N	14°52′E	Jun-13	27/33/40
Cetara	CET	30	40°37′N	14°43′E	May-13	0/87/13
Napoli*	NAP	30	40°44′N	14°17′E	Jun-13	3/63/33
Castel Volturno*	CVL	30	41°01′N	13°49′E	Jun-13	17/37/47
Sperlonga	SPL	30	41°11′N	13°26′E	Jun-13	7/60/33
Terracina	TER	30	41°14′N	13°10′E	Mar-13	0/83/17
**Ligurian and North Tyrrhenian (GSA 9)**						
Piombino	PIM	29	42°58′N	10°24′E	Dic-13	0/76/24
**Southern and Northern Adriatic (GSA 18 and 17)**						
Bari	BAR	30	41°11′N	17°05′E	Jul-13	0/73/27
Pescara	PES	30	42°54′N	14°12′E	Sept-13	10/57/33
Chioggia	CHI	30	45°08′N	12°25′E	Apr-13	7/63/30
**Western Ionian (GSA 19)**						
Cirò Marina	CIR	30	39°24′N	17°11′E	Apr-13	0/83/17
**Northern Mediterranean (GSA 6)**						
Tarragona**	TAR	29	40°53′N	01°10′E	Mar-09	0/45/55
**Atlantic Ocean**						
Cadiz** (1)	CAD1	26	36°54′N	06°16′W	Jun-12	60/0/40
Cadiz** (2)	CAD2	30	36°48′N	06°21′W	Oct-12	50/0/50
Canary**	CAN	23	27°43′N	15°39′W	May-07	39/0/61
Bay of Biscay**	BISC1	29	47°20′N	03°23′W	Sept-11	0/76/24
Bay of Biscay** (C)	BISC2	27	46°33′N	01°58′W	May-08	44/0/56
Bay of Biscay** (C)	BISC3	28	46°07′N	01°46′W	Apr-10	89/4/7
Bay of Biscay**	BISC4	30	45°52′N	01°52′W	May-08	0/90/10
Bay of Biscay** (C)	BISC5	28	45°30′N	00°56′W	Sept-11	79/14/7
Bay of Biscay** (C)	BISC6	22	45°27′N	01°12′W	Sept-09	86/0/14
Bay of Biscay**	BISC7	30	45°30′N	01°26′W	May-12	0/77/23
Bay of Biscay**	BISC8	28	44°53′N	01°27′W	May-12	0/79/21
Bay of Biscay**	BISC9	29	44°38′N	01°36′W	May-10	0/79/21
Bay of Biscay**	BISC10	28	43°22′N	02°28′W	May-08	0/82/18
Bay of Biscay** (C)	BISC11	30	43°21′N	03°03′W	Sept-10	43/7/43
Bay of Biscay**	BISC12	30	43°49′N	03°13′W	Sept-10	0/70/30
Bay of Biscay**	BISC13	29	45°38′N	02°06′W	May-12	0/90/10
Bay of Biscay**	BISC14	30	43°40′N	03°39′W	Sept-99	0/83/17
Bay of Biscay**	BISC15	30	43°38′N	05°14′W	Apr-12	0/93/7
Bay of Biscay**	BISC16	29	43°42′N	07°35′W	Sept-11	0/85/15
**EGGS**						
**Central Tyrrhenian (GSA 10)**						
Pisciotta/Capo Palinuro	E-PISC	5	40°06′N	15°12′E	Jun-13	0/20/80
Sele	E-SELE	16	40°25′N	14°53′E	Jul-13	0/69/31
Amalfi	E-AMAL	16	40°35′N	14°36′E	Jul-13	0/60/40
Capri	E-CAPR	4	40°33′N	14°17′E	Jun-13	50/25/25
Torre del Greco/Sarno	E-TGR	9	40°46′N	14°21′E	Jun-13	29/14/57
Napoli	E-NAP	19	40°47′N	14°13′E	Jun-13	13/25/63
Ischia	E-ISCH	16	40°45′N	13°51′E	Jun-13	0/78/22
Volturno	E-VOL	29	40°58′N	13°52′E	Jun-13	10/52/38
Formia	E-FORM	4	41°15′N	13°15′E	Jun-13	0/100/0

*Sites sampled for zooplankton analysis; **Montes *et al*.^20^, (C) Considered as coastal in Montes *et al*. ^20^^.^

**Figure 1:**
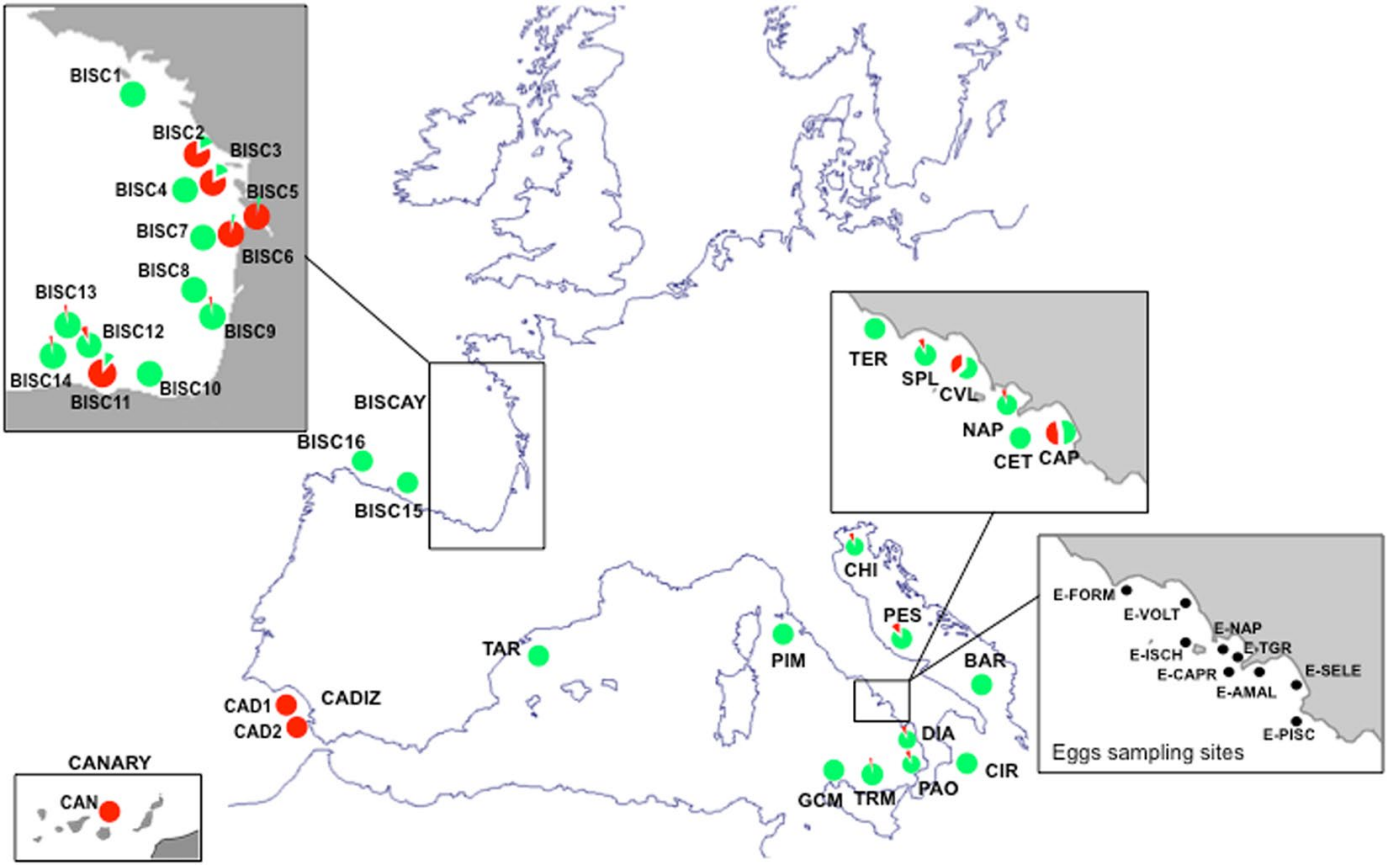
Position of the sampling sites utilized in the present analysis. Proportion of offshore (green) and the coastal (red) ecotypes is shown for each site. Sites sampled for anchovy eggs are shown in the lower right panel. Detail of populations sampled in the Bay of Biscay by Montes *et a*l.^20^ is shown in the upper-left panel. Assignment to the coastal and offshore ecotypes by Montes *et al*.^20^ is given in Table 1. The map was generated using QGis software v.2 (Quantum GIS Development Team, 2013) and modified by authors using Powerpoint software (2013) www.microsoft.com.

A Bayesian analysis of population structure, performed after including representative samples from the Atlantic coastal (narrow-shelf: Bay of Biscay coastal area, Cadiz and Canary) and offshore (wide-shelf: Bay of Biscay offshore area) ecotypes (as defined in Montes *et al*.^20^), identified two genetic groups (Fig. S1). According to assignment probabilities (AP) > 90%, we estimated the proportion of genotypes belonging to one of the two groups, in each sampling site (Fig. 1). Individuals with assignment probabilities between 50% and 90% were identified as putative hybrids. Percentages of pure and putative hybrid genotypes for each sampling site are shown in Table 1. The same analysis performed with only outliers *loci* provided very similar results (data not shown).

The coastal ecotype was highly represented in Cadiz and Canary samples and in five samples from the Bay of Biscay (sites BISC2, BISC3, BISC5, BISC6 and BISC11; Table 1, Figs 1 and S1)., In the Mediterranean Sea, the coastal ecotype was more represented in samples from river mouths in the Tyrrhenian Sea (CAP and CVL) and in the Adriatic Sea (PES), but was present with lower percentage also in other localities (TRM, PAO, DIA, NAP, SPL and CHI). Most of the Mediterranean samples harboured primarily genotypes belonging to the offshore ecotype, which characterizes all the Biscay offshore samples (Table 1, Figs 1 and S1). The two clusters co-existed in most of the sampled sites, except GCM, PIM BAR, CET, TER and CIR, where only the offshore cluster was present. The isolation of the two river-mouth sites of the Tyrrhenian Sea was supported by the analysis performed in BARRIER^23^, which identified major genetic discontinuities between samples from the CAP and CVL sites and those from all the other adjacent sites (Fig. S2).

To identify the role of local selection and adaptation in the differentiation between coastal and offshore populations along the Italian coasts, we searched for outliers in the screened SNPs panel. Bayescan software detected seven putative outliers *loci* (Fig. S3a) and fourteen were identified by Lositan (Fig. S3b). The seven *loci* common to the two approaches were identified as candidates for directional selection. Six of them were among the sixteen outlier *loci* underlying genetic differentiation of the Bay of Biscay offshore and coastal populations^20^ (Table 2). No gene functions clearly related to estuarine-low salinity environments were recorded. Two of the common outliers were related to reproduction, and in particular to ovarian follicle development (BSG – basigin) and embryo development (RPL5A – ribosomal protein L5).

**Table 2:** List of common outlier loci detected in the Mediterranean Sea by Bayescan and Lositan softwares. The first 6 *loci* overlap with outlier *loci* detected by Montes *et al.* ^20^.

Outlier markers	*F*_*ST*_		Gene/blast hit	Biologicalprocess
	Bayescan	Lositan		
*ss748771099*	0.09	0.14	RPL5A (ribosomalprotein L5)	Embryodevelopment (GO:0009790)
				Translation (GO:0006412)
*ss748771181*	0.08	0.14	TIMM10 (translocase of innermitochondrial membrane 10)	Chaperone-mediatedproteintransport (GO:0072321)
				Cellular proteinmetabolicprocess (GO:0044267)
				Mitochondrialinner membrane protein import (GO:0045039)
				Sensoryperception of sound (GO:0007605)
*ss748771203*	0.10	0.15	coiled-coil domain-containingprotein 113-like	Cell projectionorganization (GO:0030030)
				Ciliumassembly (GO:0042384)
*ss748771018*	0.08	0.15	BSG (basigin)	Pyruvatemetabolicprocess (GO:0006090)
*ss748771410*	0.08	0.12	CHAF1A (chromatinassemblyfactor1subunit A-like)	Transcription DNA-dependent (GO:0006351)
				Response to DNA damagestimulus (GO:0006974)
*ss748770985*	0.07	0.08	N/A	N/A
ss748771005	0.07	0.10	N/A	N/A

Marker names are expressed as NCBI accession numbers. *F*_ST_: overall locus-specific genetic divergence.

**Figure 2:**
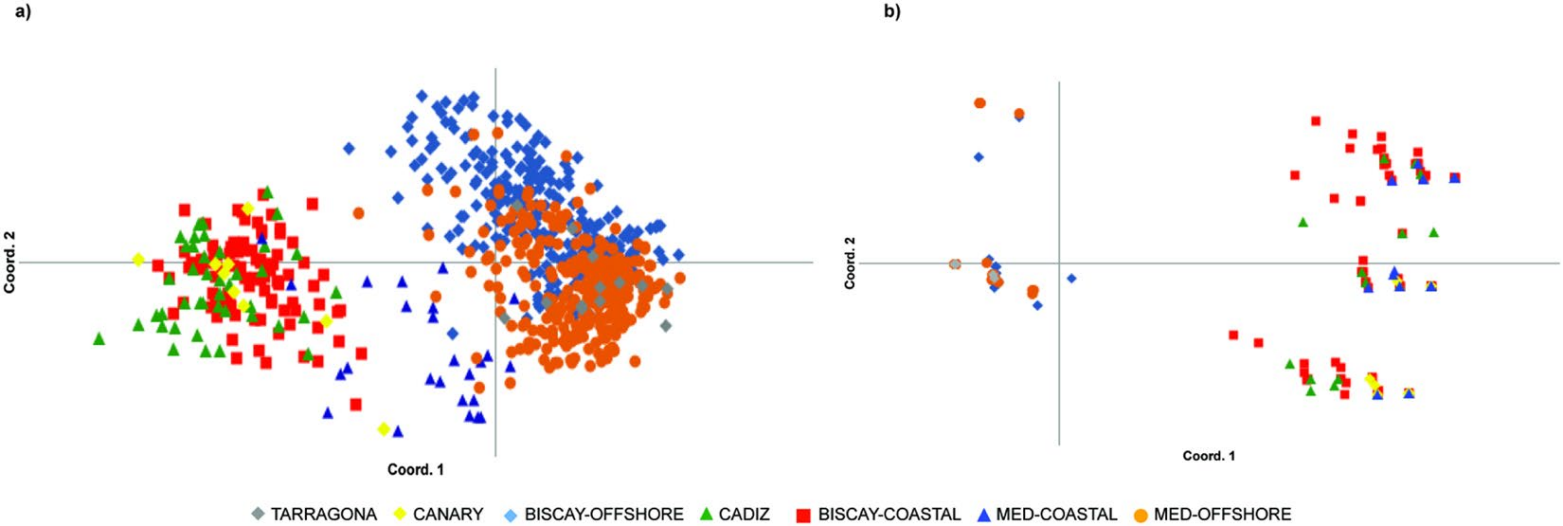
PCoA based on genetic data of coastal and offshore adult anchovies from Mediterranean and Atlantic. Putative hybrids were excluded from the analysis. (**a**) Only putative neutral markers (variance of Axis 1: 12.5%; variance of Axis 2: 4.1%); (**b**) Only putative outlier markers (variance of Axis 1: 82.7%; variance of Axis 2: 3.6%).

To assess population dispersal and connectivity among sites, excluding bias of local adaptation, we performed a PCoA excluding *loci* identified as putative outliers and putative hybrid individuals. Mediterranean and Biscay individuals were labelled according to the two Bayesian groups identified in Fig. S1. Samples from the Bay of Biscay identified as offshore by Montes *et al*.^20^ coherently grouped with the Mediterranean offshore individuals, with large overlapping (Fig. 2a). Clustering of Mediterranean coastal samples with Atlantic ones was less evident (Fig. 2a). The PCoA analysis performed only with outlier markers showed a much more evident clustering of Mediterranean and Atlantic offshore and coastal samples (Fig. 2b). When the same analysis was made including single locations it is evident that the offshore Atlantic populations strictly cluster with the Mediterranean populations that harbor an higher proportion on the offshore ecotype. The two Mediterranean populations with higher proportions of the coastal ecotype (i.e. CAP and CVL) locate toward the coastal Atlantics, and their nearness increases when using only outlier markers (Fig. S4a,b). Within the Atlantic coastal populations group, Cadiz and Canary ones are distinct from the Bay of Biscay along the second axis. To further investigate this pattern, we performed a PCoA analysis on the population pairwise mean genetic distance, highlighting the presence of two groups within the coastal ecotype: Mediterranean and Biscay coastal anchovies grouped together (Ecotype-2a), while Cadiz anchovies grouped with the Canary ones (Ecotype-2b; Fig. S4c,d). Ecotype 1 harboured offshore samples from the Atlantic Ocean and the Mediterranean Sea (Fig. S4c,d). The *F*_ST_ pairwise comparisons among all groups corroborated results of the PCoA and allowed for the detection of highly significant (P < 0.001) values of differentiations (Table 3). Values were much higher when considering only the outliers *loci*.

**Table 3:** Pairwise *F*_ST_ values calculated among Atlantic and Mediterranean (MED) offshore and coastal populations as identified by STRUCTURE, using only neutral SNPs (top triangle) and putatively outlier SNPs (bottom triangle).

	BISCAY-offshore	BISCAY-Coastal	CADIZ	CANARY	MED-Coastal	MED-offshore	TARRAGONA
**BISCAY-offshore**		**0.19095**	**0.26182**	**0.31552**	**0.15901**	**0.03523**	**0.03259**
**BISCAY-Coastal**	**0.93254**		**0.09132**	**0.20959**	**0.12809**	**0.21018**	**0.22828**
**CADIZ**	**0.96331**	**0.0489**		**0.03408**	**0.15785**	**0.26554**	**0.27291**
**CANARY**	**0.9802**	**0.16238**	*0.07527*		**0.20349**	**0.30989**	**0.33887**
**MED-Coastal**	**0.97466**	*0.05989*	**0.12338**	**0.3373**		**0.12285**	**0.12957**
**MED-offshore**	0.00167	**0.93273**	**0.96134**	**0.97666**	**0.97215**		0.00155
**TARRAGONA**	−0.01507	**0.82914**	**0.88186**	**0.94754**	**0.91159**	−0.00779	

Values in italics are significant at P < 0.05 and values in bold font are significant at P < 0.001.

**Figure 3:**
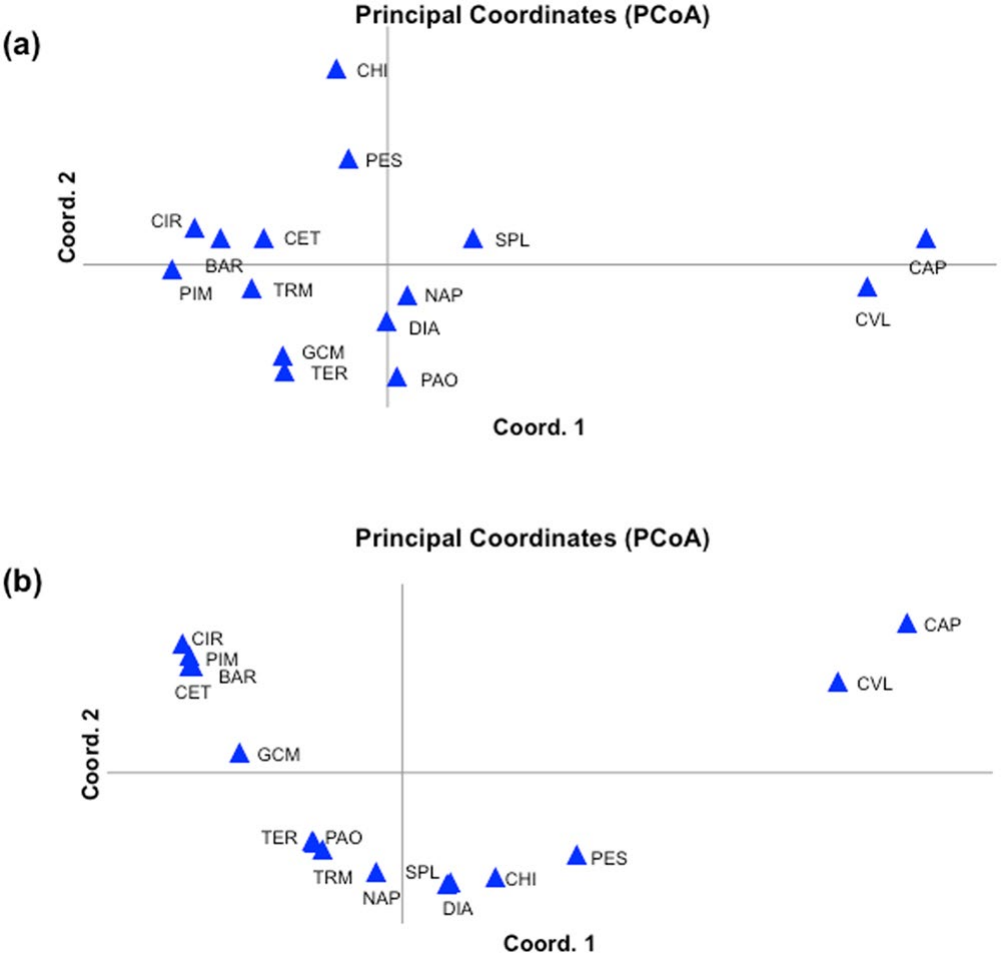
PCoA of Mediterranean anchovy samples, based on SNPs data using: (**a**) Only putative neutral markers (variance of Axis 1: 21%; variance of Axis 2: 12%); (**b**) Only putative outlier markers (variance of Axis 1: 81%; variance of Axis 2: 10%).

Finally, in order to better establish relationships among Mediterranean sampling sites, we also calculated pairwise *F*_ST_ values and the relative PCoA only for the Mediterranean Sea samples, separating neutral markers and putative outlier *loci*. The PCoA with neutral markers identified a group formed by the two central-Tyrrhenian samples collected in the proximity of river mouths (CAP and CVL) as the most distinct one along the first axis, which explains 21% of the total variance. Al the other samples cluster toward the negative side of the first axis. The two Adriatic samples from PES and CHI were slightly differentiated along the 2nd axis (variance,12%; Fig. 3a). The PCoA with outlier markers showed a more continuous gradient along the first axis (variance, 81%), from sampling sites with the majority of offshore genotypes to sampling sites with the majority of coastal ones (Fig. 3b). CAP and CVL are more isolated from the others. The pairwise *F*_ST_ comparisons confirm this pattern (Table 4).

**Table 4:** Pairwise *F*_ST_ values calculated among Mediterranean sampling sites of adults and eggs, using putatively neutral (top triangle) and putatively outlier SNPs (bottom triangle).

	BAR	CAP	CET	CHI	CIR	CVL	DIA	GCM	NAP	PAO	PES	PIM	SPL	TER	TRM	E-AMAL	E-ISCH	E-NAP	E-SELE	E-VOLT
BAR		0.02191	0.01317	0.00713	0.00365	*0.01571*	0.00244	−0.00343	−0.00216	*0.01089*	−0.00269	0.00175	−0.00138	0.00361	−0.00542	0.0005	0.00233	0.04301	0.01051	0.01955
CAP	0.39433		0.03561	0.02902	0.03191	−0.00682	*0.01914*	*0.02234*	*0.01405*	*0.02403*	*0.01944*	0.03221	0.00869	0.02772	0.02376	0.01761	0.01118	−0.01064	0.00581	−0.00853
CET	0.01124	0.40778		0.02569	0.023	*0.01921*	0.0242	*0.01693*	*0.00985*	0.02214	0.01095	0.0054	*0.01168*	0.019	*0.00946*	*0.03065*	0.01223	0.04327	0.02528	0.031
CHI	*0.0974*	*0.15973*	0.09135		0.02028	*0.01953*	0.02401	0.01766	0.00813	0.02578	−0.00031	*0.01059*	*0.0113*	0.02494	*0.0137*	*0.01378*	*0.01773*	0.04698	*0.01689*	0.03151
CIR	0.00385	0.42534	0.01961	0.11409		0.02953	0.01646	*0.00894*	*0.01001*	*0.01627*	0.00473	0.01436	*0.01409*	0.01804	*0.00715*	*0.01744*	0.00919	0.05842	0.02028	0.03176
CVL	0.36136	−0.02071	0.37286	0.10676	0.39611		0.00949	0.01148	0.00128	0.00671	0.01262	*0.01591*	0.00403	*0.01436*	0.00751	0.00328	0.00889	−0.01178	−0.001	−0.00998
DIA	0.07799	*0.18763*	0.07953	−0.01819	*0.09818*	*0.13615*		0.00356	0.00144	−0.00031	0.00098	0.00398	0.00042	0.00134	0.00108	0.00069	0.00225	0.04217	0.00754	*0.01326*
GCM	0.00179	0.34579	−0.00618	0.05187	*0.02319*	*0.3037*	0.03683		−0.00458	−0.00146	0.00801	0.00208	0.0028	−0.00007	−0.00353	0.00452	0.00369	0.04026	*0.01486*	*0.01707*
NAP	*0.04394*	*0.23886*	0.03969	−0.0087	*0.06031*	*0.18774*	−0.01699	0.00553		0.00162	0.00172	−0.00416	−0.00157	0.00448	0.00097	0.00601	−0.00468	*0.0266*	0.0049	0.01192
PAO	0.01967	0.28937	0.0183	0.01839	0.03201	0.24344	0.00176	−0.00454	−0.0155		0.00864	*0.01337*	0.00706	0.0026	0.00344	0.00547	*0.01639*	0.04775	*0.01364*	0.017
PES	0.13582	0.10329	0.13594	−0.01636	*0.15503*	0.05631	−0.01191	0.08942	0.01435	0.04511		0.00516	−0.00135	*0.01102*	0.00175	0.0033	0.00085	0.04476	0.00523	*0.0167*
PIM	0.00000	0.39401	−0.01726	*0.09378*	*0.00385*	0.36036	*0.08096*	0.00179	*0.04394*	0.02295	0.13481		0.00206	0.00494	−0.00156	0.00771	0.00028	0.04715	0.01299	0.029
SPL	0.07808	*0.18743*	0.07464	−0.01666	0.09419	*0.13461*	−0.0227	0.03639	−0.0135	0.00317	−0.0117	0.07808		0.00686	−0.00053	0.00051	−0.00684	0.0145	0.00259	0.00627
TER	0.01982	0.30057	0.01563	0.01469	0.0298	*0.25245*	0.00142	−0.00639	−0.01683	−0.0193	0.04297	0.01982	0.00035		−0.0006	−0.00095	0.00727	0.04403	*0.01252*	0.01796
TRM	0.02366	0.28314	0.02331	0.01167	0.03536	*0.23573*	−0.00387	−0.00298	−0.01806	−0.0205	0.03564	0.02645	−0.00448	−0.02108		−0.0002	0.00096	0.04251	0.01077	*0.01295*
E-AMAL	0.23664	0.04855	*0.24331*	−0.00837	*0.27295*	0.009	0.00091	0.16192	0.04391	0.09415	−0.03168	*0.23664*	0.00236	0.09148	0.07843		−0.00638	*0.04201*	0.00199	0.01114
E-ISCH	*0.17457*	0.1024	*0.17886*	−0.03267	*0.21768*	0.05732	−0.0331	0.09013	−0.00926	0.03011	−0.03565	*0.17789*	−0.026	0.0283	0.0201	−0.03685		*0.02948*	−0.00559	0.00732
E-NAP	0.49561	−0.02953	0.51098	*0.21938*	0.53029	−0.00972	*0.25363*	0.43889	0.31428	0.37486	*0.15147*	0.49529	*0.25307*	0.38514	0.36564	0.08537	*0.15413*		0.00899	−0.00764
E-SELE	*0.26138*	0.02053	0.27143	0.02716	*0.29239*	−0.00881	0.04008	*0.20173*	0.08941	*0.13826*	−0.0094	*0.26138*	0.0406	*0.13905*	0.12414	−0.03924	−0.00871	0.04648		−0.00082
E-VOLT	0.2326	0.01348	0.24014	0.04118	0.25559	−0.01186	0.0548	*0.1891*	0.09789	*0.13772*	0.00521	0.2326	0.05598	0.14166	*0.129*	−0.02513	0.00519	0.03791	−0.02954	

Values in italics are significant at P < 0.05 and values in bold font are significant at P < 0.001.

Though all anchovy egg samples were successfully genotyped, we eliminated from the analysis the sampling sites with negligible egg occurrence (<16 eggs) (E-PISC, E-CAPR, E-TGR, E-FORM). When the other egg samples were included in the PCoA with neutral *loci* only, three sites (E-NAP, E-VOLT and E-SELE) clustered together with Tyrrhenian samples from river mouths (CVL and CAP) in the positive side of the first axis (Fig. S4c). Using only outlier markers, the eggs showed a gradient similar to that of adult anchovies (Fig. S4d).

### Environment and diet

The genetic analyses indicated that, despite the large environmental differences, one of the coastal ecotypes (ecotype-2a) is largely similar between the Mediterranean Sea and the near Atlantic. To test the hypothesis that behaviour and not environmental factors are at the origin of the separation of this group, we carried out supplementary analysis at local scale using a multidisciplinary approach.

**Figure 4:**
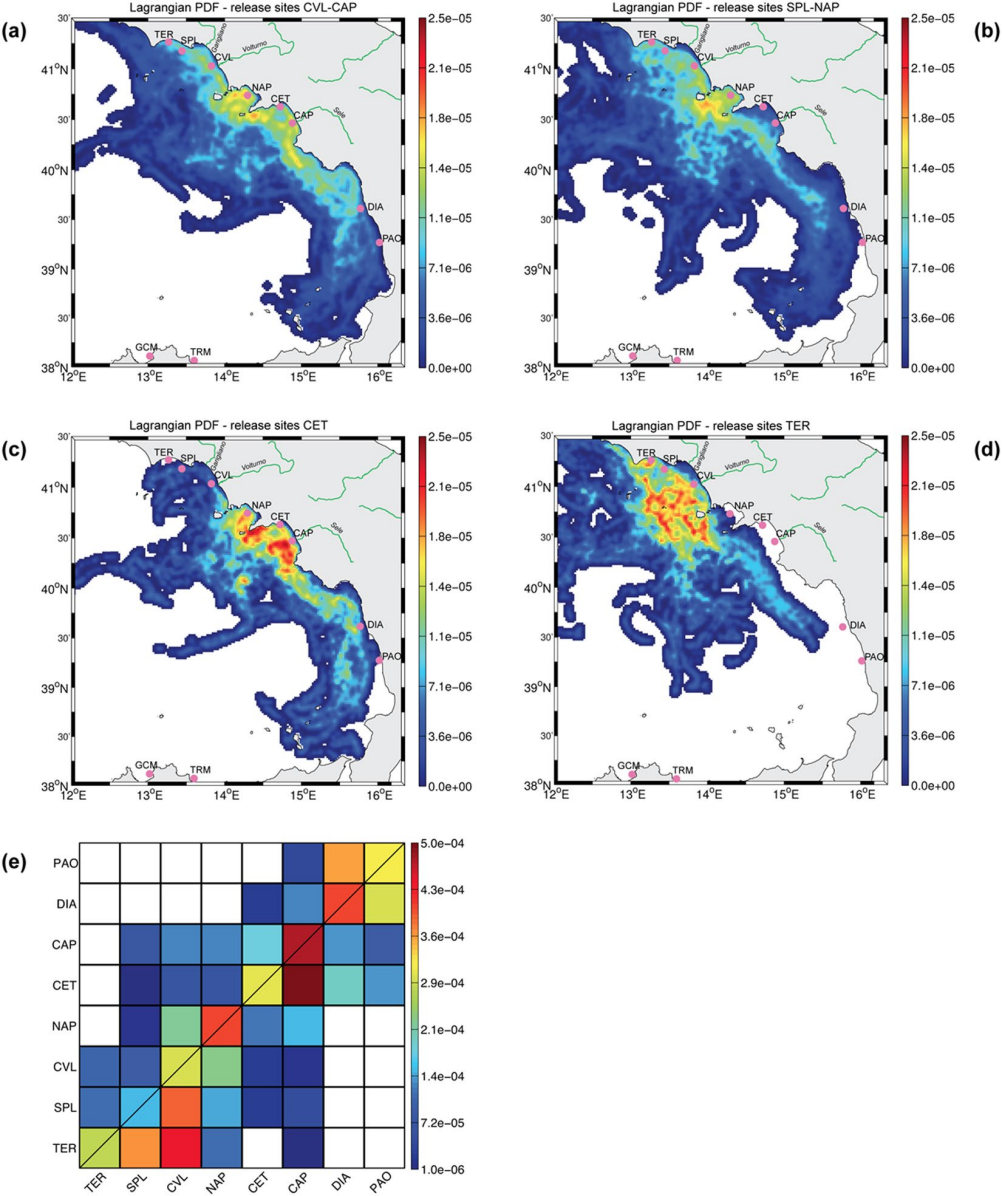
Results of the Lagrangian analysis. (**a–d**) Lagrangian Probability Density Functions of particles presence after an advection time of 30 days for four different released sites selected along the Tyrrhenian coast. LPDFs for CVL/CAP (or SPL/NAP) are inferred from the combined data when particles are released both in CVL and CAP (of SPL and NAP respectively). (**e**) Connectivity matrix between Tyrrhenian sites, where adult anchovies were collected. Each box gives the probability that a particle released in a region in X-axis reaches a region in Y-axis. Thus, a value of 10^4 means that over 10^4 released particles, one particle reached the site of interest. Results show that even though the Gulf of Naples exhibit a strong retention power, no preferential areas exist along the Tyrrhenian coast. The small probability of connectivity for long distanced sites is to be mitigated by the fact that billions of larvae are advected in the sea. Thus, dispersal can not explain the genetic separation between the two ecotypes. Maps in Fig. 4 were generated using MATLAB software Version 7.7.0.471 (R2008b); www.mathworks.com.

First, we investigated the presence of mechanisms that may favour a physical separation between sampling sites by evaluating the local physical connectivity, i.e., how dispersal processes connect the different coastal regions of the Tyrrhenian Sea. Excluding the active dispersal of adult anchovies, we simulated egg dispersal only during the months of their occurrence and in the areas where adults were collected. We conducted a series of simulations of passively advected point-like particles released from sampling sites during May-August for three different years (2009, 2012 and 2013, details in Materials and Methods). The trajectories were then used to infer the most probable particle density in the sea 30 days (a proxy for the recruitment time scale) after the release from a given site. Results showed that particles released at CVL/CAP (Fig. 4a) and SPL/NAP (Fig. 4b) can potentially reach all the central-southern Tyrrhenian coasts. In contrast, particles released at CET and TER have a higher probability to be dispersed toward the southern and northern Tyrrhenian Sea, respectively (Fig.4c,d). Connectivity among sampling sites for adult anchovies is summarized in Fig.4e. Overall, connectivity is present among all sites except for the southernmost sites of DIA and PAO.

**Figure 5:**
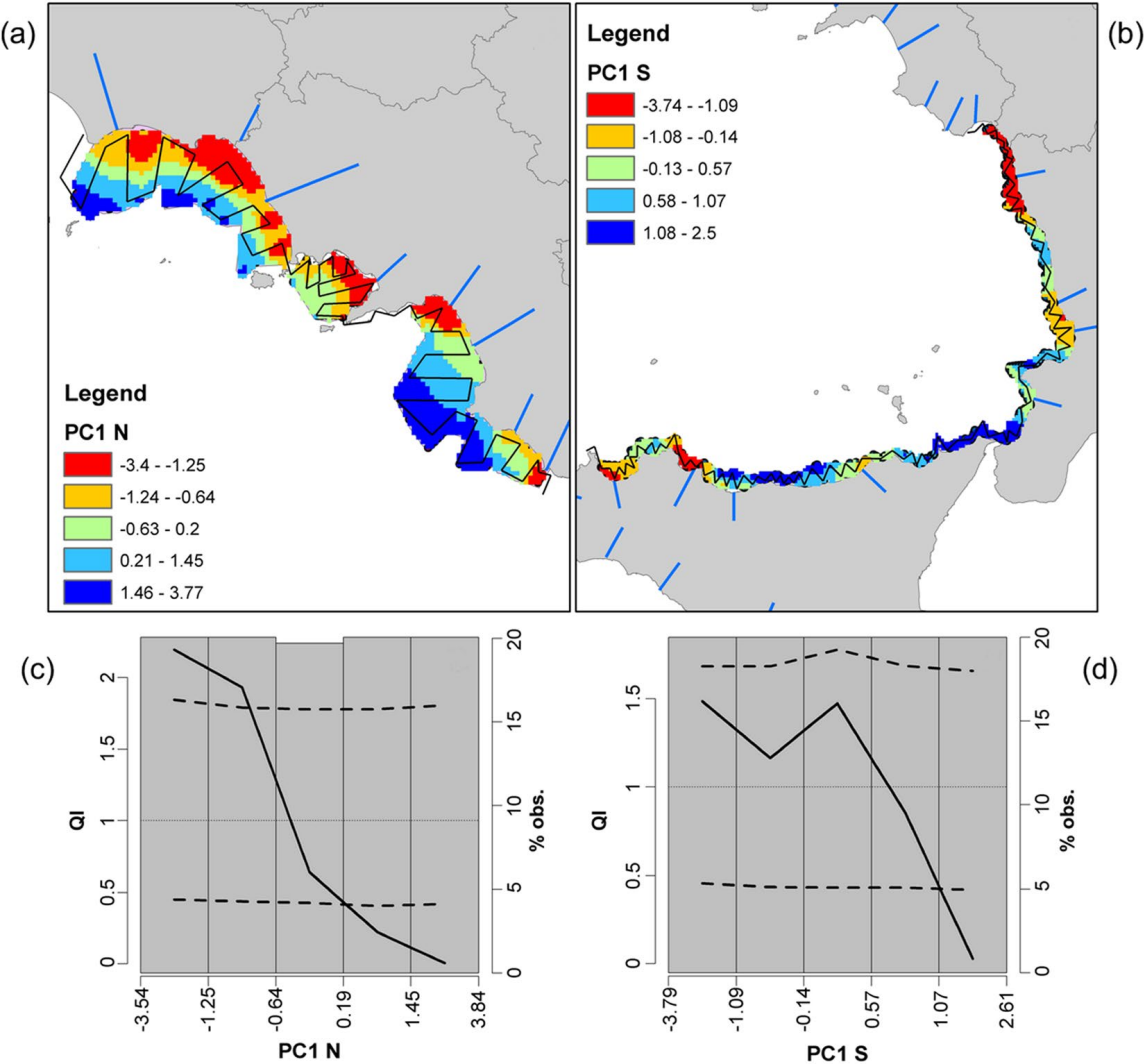
Environmental correlation of anchovy abundance. Top panels: spatial representation of PC1 scores in the Northern (**a**) and Southern (**b**) areas of the Central-Southern Tyrrhenian Sea. Colours represent PC1 scores, according to the ranges identified in QI analysis (bottom panels; c and d). In the northern sector the red and orange sectors represent the most “favourable” areas for anchovy, while the light blue and blue ones represent avoided sectors. Black lines represent the acoustic survey design. Bottom panels: QI analysis results, highlighting the response (selection, avoidance, tolerance) of anchovy population to the identified environmental processes (PCA) in the Northern (**c**) and Southern (**d**) areas. Dashed lines identify the upper and lower confidence intervals of the QI curve (solid line). QI values higher than 1 and above the upper confidence interval identify a “selective” behaviour, while QI values lower than 1 and below the lower confidence interval indicate “avoidance” behaviour. “Tolerance” behaviour is in between. Maps in Fig. 5 were generated using QGis software v.2 (Quantum GIS Development Team, 2013).

Second, we performed an analysis of the environmental variables, which showed a clear difference between the northern and southern sectors of the central/southern Tyrrhenian basin. The PCA, carried out separately for the two sectors (Table 5), showed a dominant pattern (the first axis accounting 47% of total variability) in the Northern part (from CAP to TER, Fig. 1), linking higher productivity to lower salinity, depth, and distance from river mouth. Furthermore, in this area, anchovy biomass was strongly associated to the river runoff (Fig. 5) and, according to the prominent environmental features, higher abundance of the coastal ecotype was recorded (Fig. 1). Conversely, in the southern area, a less clear picture emerged about the dominant environmental patterns driving the environmental variability and their relations with the relative abundance of the two anchovy ecotypes. The PC1 in the southern part explained a lower percentage of the total variability (about 35%; Table 5) and was strongly related to temperature only (describing probably a pure physical process), while the PC2 highlighted higher productivity in coastal sectors. In the southern sector, characterized by a weak effect of river runoff (Fig. 5), a higher percentage of the offshore ecotype was recorded (Fig. 1).

**Table 5:** Values of significant (P < 0.05) correlations among considered environmental variables and 1^st^ and 2^nd^ PCs axes.

	Northern area		Southern Area	
	PC 1 (47%)	PC 2 (25.9%)	PC 1 (35.5%)	PC 2 (23.6%)
Chl-a (mg/m^3^)	−0.801	0.093	−0.454	−0.614
River distance (Km)	0.798	0.271	0.468	0.263
Depth (m)	0.692	0.482	0.109	0.743
Temperature (°C)	−0.166	0.884	−0.823	0.318
Salinity (PSU)	0.747	−0.44	0.81	−0.274

**Figure 6:**
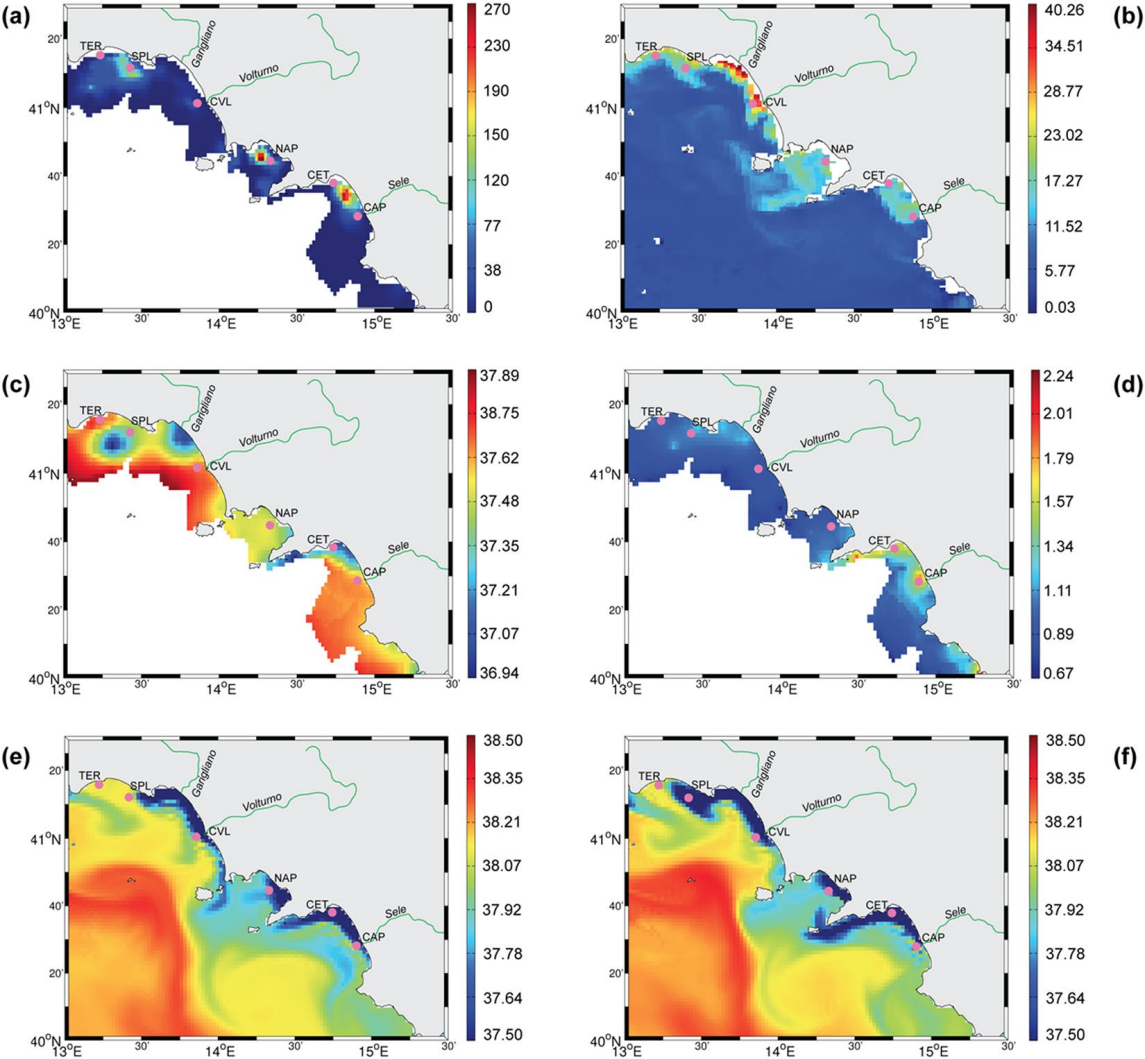
The oceanography data and anchovy biomass distribution on the days July 5-7 2013 (EVATYR13), when sampling in the area was performed. (**a**) anchovy biomasses on July 6–7 2013; (**b**) Surface Chl from satellite (July 6). (**c**) surface salinity from CTDs; (**d**) water transparency from CTDs; (**e**) modelled surface salinity from ROMS for July 7^th^; (**f**) Surface salinity from ROMS for the July 5^th^. Maps in Fig. 6 were generated using MATLAB software Version 7.7.0.471 (R2008b); www.mathworks.com.

Third, to further investigate how the presence of river mouths can affect population fine-scale patterns, we analysed the oceanographic conditions in a small area north of the Gulf of Naples, including the Volturno river. Acoustic survey showed that the highest anchovy biomasses were recorded in Sperlonga, in the Gulf of Naples and in the Sele River plume (Fig. 6a). Satellite images showed that anchovies from SPL were caught in an area occupied by a coastal front characterized by high chlorophyll (Chl) (Fig. 6b) and low salinity, which originated from the Volturno River runoff (CTD data in Fig. 6c,d). Looking at the simulated salinity fields, we could trace the plume backward in time and show that it originated from the accumulation on the shelf of Volturno River waters (Fig. 6e,f). Hence, both SPL and CVL anchovy populations (see Fig. 1 for the position of the two stations) can be associated to the Volturno River. While the SPL anchovies were following a high productivity plume created by the river and moving north (see Fig. 6e,f), most of the coastal ecotype fishes, hanged around the front of the river mouth (in CVL) despite the fact that most of the river-associated productivity had displaced elsewhere in the previous days (Fig. 6f).

Fourth, we assessed that the diet of adult anchovies was almost exclusively based on zooplankton. Results showed that only 0.5% of prey number were represented by phytoplankton cells (Table S1). The clustering based on diet composition clearly separated the two anchovy samples with higher percentage of the coastal ecotype (CAP and CVL) from the others (Fig. S6a,b), matching very well the genetic separation. The diet of anchovies in the two estuarine areas was mainly characterized by small-sized copepod genera (prosome length < 1 mm), like *Euterpina, Farranula, Oithona, Acartia*, and other calanoids and their nauplii, together with other small-sized prey like bivalve larvae. The stable isotope analysis of adult anchovies (>8.5 cm Total Length) revealed values of δ^15^N and δ^13^C from 6.61‰ to 11.91‰ and from −17.07‰ to −13.24‰, respectively. The δ^15^N values showed a clear geographical pattern with an increasing trend northward, although δ^15^N values in SPL were slightly lower than in CVL and NAP (Fig. S7a). The δ^15^N isotopic signal presented significant differences (P < 0.05) between the areas GCM/PAO/CAP and NAP/CVL/SPL. Differences of δ^13^C were not significant, although higher values were detected in NAP samples (Fig. S7b). The remarkable spatial heterogeneity highlighted by the diet and trophic position of adult anchovies did not appear from the distribution of their prey. In fact, zooplankton communities, dominated by copepods (84.0 ± 5.3%) during the survey (Fig. S5b,c), differentiated only at station GCM (Fig. S5c), as a result of the highest total abundance and the dominance of the coastal species *A. clausi* (76.4%).

## Discussion

We analysed the genetic diversity among populations of *Engraulis encrasicolus* in the Mediterranean Sea, and we showed the existence of two genetic entities, i.e. offshore and coastal ecotypes, that coexist in most of the sampled sites and strongly resemble the two ecotypes observed on the Atlantic coasts. We therefore confirm and extend the results by Le Moan *et al*.^9^ about anchovies in the Central Mediterranean Sea using an higher number of individuals and on a wider geographic scale. Moreover, we found that the evolutionary divergence between the two ecotypes in the Mediterranean Sea is lower than in the near coastal Atlantic.

Along the central/southern Tyrrhenian coasts, the coastal anchovy ecotype has been found more abundant at two river mouths where abundance was positively correlated with lower salinity and higher chlorophyll values. In addition, anchovies sampled at the river mouth had a distinct diet characterized by small sized preys such as small copepods and bivalve larvae. The trophic position of adult anchovies showed a clear difference between the northern and the southern sectors of our sampling area in the Tyrrhenian Sea, therefore reflecting differences over a deeper time scale with respect to stomach content analysis.

Specifically, a higher trophic level for both the coastal and offshore ecotypes in the northern sector (despite the different diets at the moment of the capture) and a lower trophic level for the offshore ecotype in the southern sector suggest that the offshore ecotype adapts to local food availability, profiting of the richer environments where rivers are presents but able to explore and thrive also in more oligotrophic conditions.

Sampling sites connectivity, governed by marine currents, does not explain the genetic patterns observed, while SNPs neutrality test identified seven putative outlier *loci*, suggesting selective divergence between the two ecotypes. Overall, our observations suggest that the two anchovy ecotypes herein identified correspond to the offshore and coastal ecotypes previously described in the Atlantic Ocean for the European anchovy^6^, despite the remarkable environmental differences between the two regions. Our local-scale analysis showed that the two ecotypes can co-occur in the same localities and that populations with a higher representation of the coastal ecotype have a strong relation with the river mouths, while the other ones have more plasticity, i.e., are able to profit of river-originated productivity though generally live in more oligotrophic conditions. We did not found evidence of a specific adaptation to river environments, in the case of these very small rivers. Two of the outliers genes identified in the analysis were associated with reproductive processes, but this is not sufficient to draw any conclusion. Further analysis assessing traits under selection are necessary.

The Atlantic offshore ecotype is associated with wide continental shelves and harbours anchovy populations in the Bay of Biscay and the North Sea^20^, while that of the Mediterranean Sea is associated to regions with almost complete absence of shelf and with more than 10 °C higher temperatures, much higher salinities, and highly oligotrophic conditions. Given the large differences in environmental conditions, we propose that the common trait is high plasticity that makes this ecotype highly opportunistic when resources are available (e.g., river plumes, open ocean blooms) while being able to live in highly oligotrophic conditions. It is worth noting that offshore populations from the Bay of Biscay and the Mediterranean Sea have a lower genetic differentiation levels, in respect to the coastal populations. This is likely due to the presence of a common ancestor and to the higher dispersal conferred by higher ecological plasticity.

The Atlantic coastal ecotype is associated with narrow continental marine shelves, including populations from Iberian-Atlantic coasts and Canary Islands while in the Mediterranean Sea it seems to be associated to river mouths. However, this narrow-shelf ecotype is not as homogeneous, since Cadiz/Canary samples significantly differ from the coastal group composed by Biscayan and Italian coastal samples (Table 3; Fig. S4a,b). Coastal anchovies seem to avoid the risks of an open sea environment, showing high fidelity to river mouths, which is a more stable source of resources. In turn, this fidelity creates a much reduced gene flow among sites, thus potentially explaining why these ecotypes are more differentiated across the basins.

This result adds relevant information to the observations of Montes *et al*.^20^, stressing that Biscayan and Italian coastal populations are different from Cadiz/Canary narrow-shelf populations, but most probably share a common origin.

Atlantic and Mediterranean populations of coastal and offshore ecotypes may have become geographically isolated from each other, resulting in an allopatric divergence followed by secondary overlap (see also Le Moan *et al*.^9^). These ecotypes could have diverged in different *refugia*, i.e., wide continental and narrow/non-existent continental shelves^20^, during the last glaciation event (LGE). Then, they could have extended their distribution in different directions, once temperature started to arise. The coastal ancestor migrated from the Atlantic and Mediterranean *refugia* to estuaries and areas with river inputs, in the two basins, even when these areas were already inhabited by wide-shelf populations. If genomes of the two different ecotypes evolved to incompatibility during allopatry, no (or very little) gene flow would have been possible upon secondary overlap and ecotypes of the same area would have remained genetically distinct^24^.

An alternative hypothesis is that a sympatric or parapatric speciation due to divergent selective pressures promoted the evolution of different ecotypes along with their partial reproductive isolation even if present in the same area. Favourable habitats or environmental constraints (e.g. rivers and lagoons) could influence the growth of individuals and development of stocks on the basis of their genetic adaptability. Barriers to gene flow between the two ecotypes could have evolved in a short time because divergence driven by ecological separation requires minor genetic alterations only in few *loci* ^25–28^. Mixed hauls of the two ecotypes were found both in the Bay of Biscay^20^ and Tyrrhenian Sea areas, although the segregation of the two ecotypes is stronger in the Atlantic populations. A lower amount of putative hybrid individuals was also detected in the Bay of Biscay in respect to most of the Mediterranean populations, suggesting stronger reproductive isolation in this basin. As previously suggested, in the Mediterranean Sea genomes of the two ecotypes could be differentiated in regions harbouring local adaptation, but can be more homogeneous in other regions, due to insufficient divergence or secondary contact by gene flow^6^. The putative congeneric species *E. albidus* ^13, 14^ and the putative subspecies *E. encrasicolus russoi* ^29, 30^ could be the result of similar speciation processes.

Searching for putative outliers in separating coastal and offshore Mediterranean populations, we identified 7 *loci* among the whole panel of SNPs utilized. Surprisingly and interestingly, 6 of them are among the 16 outlier *loci* that are involved in differentiation of the Bay of Biscay coastal anchovy^20^. This finding that the 37.5% of outlier *loci* separating the two ecotypes in the Bay of Biscay also separate the two ecotypes in the Mediterranean, is higher than the value of 24.1% found by Le Moan *et al*.^9^. Nevertheless, it strongly confirms the complexity of the evolutionary origin of the two ecotypes and the possibility that the distinction by functional *loci* has arisen when the two common ancestors started to separate. The fact that the basigin gene, involved in the fusion of egg and sperm, appears as a common outlier differentiating coastal and offshore populations in both Mediterranean Sea and Bay of Biscay, suggests a selection pressure on reproduction-related genes that could reduce gene flow between these ecotypes on a wider scale. On the other side, the separation observed between ecotypes in the two basins, also when outlier *loci* are excluded from the analysis, suggests that neutral genetic drift cannot be neglected.

The coexistence of parental populations and hybrid hauls in the same areas, and the apparent patchiness in hybrids distribution could be an effect of larval transport to areas distant from the areas of fertilization^31^. The position of spawning areas is genetically fixed and those occur in optimal environmental conditions for survival of offspring. However, distribution and abundance of eggs and larvae are primarily affected by passive transport^2, 32^, and our data suggest high rate of eggs dispersion along the Italian Tyrrhenian coasts. Patterns of marine currents affect the distribution of small pelagic fishes also by shaping the distribution of their prey, most of which are unable to move for long distance^33, 34^. The results of the Lagrangian analysis showed that in Central Tyrrhenian Sea the geomorphological conformation of gulfs favours retention of the nutrient-rich river waters, thus amplifying the impact of rivers that are actually very small (flow < 100 m^3^/s), especially if compared with the rivers along the Bay of Biscay.

In conclusion, we shed light on the existence of two anchovy ecotypes co-occurring along the European coasts: the offshore ecotype, with wide distribution in the North Eastern Atlantic Ocean and in the Mediterranean Sea, and the coastal ecotype with preferences for river plumes. These ecotypes show signs of evolutionary divergence at pan European scale, although their separation in the Mediterranean Sea is lower than in the near Atlantic Ocean. Their co-existence in the same populations and the presence of many hybrids make difficult their inclusion in the stock management plans. Further studies are necessary to address the question whether such genetic differences could lead to differences in reproductive and recruitment success or in other related factors influencing the abundance and spatial distribution of the two groups.

## Methods Summary

### Sample collection

A total of 443 anchovies from 15 sites were collected from the Tyrrhenian, the Adriatic and the Ionian Sea (Table 1). The adult anchovies were caught by midwater pelagic trawl net (78 m of length and 18 mm of mesh) equipped with Simrad ITI sound system for the geometry of the net control during sampling. Samples were immediately frozen on board (−20 °C).

We also collected eggs of *E. encrasicolus* at six stations along the coasts of the South Tyrrhenian area (Table 1). Mesozooplankton and fish eggs were collected by oblique tows in the upper 50 m layer with a Bongo net (40 cm mouth diameter, 200 µm mesh size). Half of the mesozooplankton sample was fixed in formaldehyde-sea water solution (4%) for taxonomical analysis and the other half was immediately frozen on board (−20 °C). No use of live animals has been required for this study and no specific permissions were needed for the sampling activities in all of the investigated areas because our species of interest is commercially harvested (not endangered nor protected) and it was caught in areas where fishing is allowed.

### Genetic analysis

Total genomic DNA was isolated from 30 mg of tissue of adult anchovies using Nucleospin Tissue kit (Macherey-Nagel, Düren, Germany), following manufacturer’s protocol. Eggs genomic DNA was isolated by a Hotshot protocol (Catanese *et al*., in prep.). All individuals were genotyped for 96 SNPs^22^ using Fluidigm Biomark platform 96.96 chips (Life Technologies, Carlsbad, USA), following the user guide. The panel of 96 highly informative SNP markers was selected in a previous study where it showed optimal resolution in assessing differentiation among *E. encrasicolus* populations within the Mediterranean and between Atlantic and Mediterranean Sea^22^. SNP genotyping was performed in the Genomics Facilities-Sgiker (UPV/EHU).

Additionally, data from 565 samples from Gulf of Cadiz (CAD), Canary (CAN), Tarragona (TAR) and Biscay (Biscay-offshore and Biscay-coastal), analyzed in Montes *et al*.^20^ were also included in the analysis, and compared for the 96 SNP utilized in our analysis (Table 1). This was possible since samples were genotyped with a SNPs panel which included the 96 SNPs herein utilized.

Genetic differentiation was assessed using the program Genepop 4.1.0^35^ and a principal component analysis (PCoA) was conducted with GENALEX^36^ and the package ADEGENET v.1.3-1 for R^37^. STRUCTURE version 2.3.4^38^software was employed to infer main Bayesian genetic clustering. Runs in STRUCTURE were made assuming K = 1– 5, imposing an admixture model with correlated allele frequencies for estimating the ancestral populations and indicating the sampling location information. Each K value was replicated with ten independent runs of 100,000 MCMC iterations, after a burn-in of 10,000 iterations. The most likely number of clusters based on delta K was identified using the method of Evanno *et al*.^39^, performed in the software STRUCTURE HARVESTER^40^. Individuals with assignment probability > 90% were considered as belonging to a given cluster, while individuals with assignment probability between 50 and 90% were considered as putative hybrids.

Patterns of population differentiation among all samples (pairwise *F*_ST_ and genetic distances) were calculated by the program ARLEQUIN v.3.5^41^. P-values of *F*_ST_ values were computed using a permutation approach (2,000 iterations). BARRIER v.2.2^23^ was used with pairwise estimates of *F*_ST_ mapped onto a matrix of geographic coordinates (latitude and longitude), and the Monmonier’s maximum difference algorithm identified breaks in gene flow patterns among geographical sites. BAYESCAN v.2.1^42^ and LOSITAN^43^ were used to test whether any of the used SNP markers do not behave according to expectations under neutrality. In BAYESCAN, we calculated Q-values as posterior probabilities to estimate the difference in numbers of putative neutral and selected *loci*, performing 20 pilot runs with 5,000 iterations and 500,000 iterations MCMC with an additional 50,000 iterations as burn-in. LOSITAN is based on a coalescent approach, detecting outlier *loci* from the joint distribution of *F*_ST_ and expected heterozygosity (He) under the island model of migration. Runs were made with both the Infinite Allele Mutation Model (IAM) and Stepwise Mutation Model (SMM), each with 100,000 simulations. Only the *loci* that were detected by both methods were considered as true outliers.

### ROMS model set up

The Regional Ocean Modeling System (ROMS) is a free-surface, terrain-following, primitive equation ocean model, with online point-particles tracking abilities^44, 45^. Here, we conducted numerical simulations, using ROMS, to simulate the circulation of the Tyrrhenian Sea, with a particular attention to the effect of river discharges on the surface circulation and associated tracer distribution. Our model domain extends roughly from 8 E to 16 E in longitude and from 36.5N to 44N in latitude, with a horizontal resolution of 2 km and 30 vertical sigma-levels. Such grid resolution is sufficient to capture the scales of interest (see Iermano *et al*.^46^) from the large scale geostrophic vortices (~100kms) down to the turbulent (sub) mesoscale and wind-induced perturbations (<10 kms) and guarantees a smooth nudging with the lower resolution operational model output used to define the open boundary conditions. Initial and boundary conditions are derived from the Mediterranean Monitoring and Forecasting Centre (MED-MFC) physical reanalysis product. The MED-MFC model data are presently being produced and distributed on a weekly basis by the European Copernicus Marine Environment Monitoring Service (CMEMS, http://marine.copernicus.eu). They are obtained through a specific implementation of the hydrodynamic model supplied by the Nucleous for European Modelling of the Ocean (NEMO), including a variational data assimilation scheme (OceanVAR) for *in situ* temperature and salinity vertical profiles and satellite Sea Level Anomaly along-track data^47^. The MED-MFC data are available on a 1/16° × 1/16° horizontal resolution grid (6–7 kms here) and 72 vertical levels. Hence, since such resolution differs from that of our domain (2 kms), we project the sea surface height, the seawater velocity fields, the temperature and salinity MED-MFC data at the boundaries, and as initial condition, using a trilinear interpolation. We nudge temperature and salt in the regions close to the boundary to allow for a smooth transition from the boundary data, with a nudging linearly decreasing from a time scale of 0.25 days at the boundary to a null value at about 100 kms from the boundary.

Campania (South Tyrrhenian Italy) rivers have been monitored by the Protezione Civile (the Italian Civil Protection Agency) of the Campania Region since 2001. Observed daily discharge data were incorporated in the model as a point source term.

The air-sea interaction in ROMS is modelled using a bulk parametrization of Fairall *et al*.^48^. It was adapted from the Coupled Ocean-Atmosphere Response Experiment (COARE) algorithm for the computation of surface fluxes of momentum, sensible heat, and latent heat. The air-sea boundary layer is used as a one way coupling with atmospheric models. Here, we use the atmospheric data of the nonhydrostatic version of the SKIRON/Eta modelling system, that was implemented in the Mediterranean and Black sea in order to produce high-resolution (10km) weather hindcasts and forecasts. The surface fluxes, obtained through bulk parameterization, and the SKIRON data have been validated against the NCEP Reanalysis Data. The NCEP/NCAR Reanalysis 1 project is using a state-of-the-art analysis/forecast system to perform data assimilation using past data from 1948 to the present^49^.

In order to assess the physical connectivity among sampling sites, we ran simulations of 120 days from May to August for the years 2009, 2012 and 2013, therefore covering the late-spring/summer periods of the year when *in situ* sampling was carried out. Results obtained with ROMS appear as a smooth extension of the MED-MFC data. Figure 6e and f show the fields of salt, temperature and velocity after one day of simulation. The temperature fluctuations are consistent and comparable with previous circulation modelling of the Tyrrhenian Sea^50–52^. However, the nudging strongly conditions the results of the simulation and prevents the development of small scales non-linearities. Finally, in order to conduct Lagrangian analyses and infer the physical connectivity along the coasts of the Tyrrhenian Sea, we released and tracked a set of point-like particles being passively advected by currents. Particles are released on the surface along the coast around each sampling sites in a disk-like area with a radius of Rsite = 4 km. Starting in May and during 90 days, a batch of 550 particles is released from every site with an occurrence of 5 days for a total number of around 10,000 particles per site.

### Lagrangian analysis

From the particles trajectories computed with ROMS, we inferred the fine-grained Lagrangian PDFs which give the probability density function of a particle leaving its initial location and reaching a given destination location of interest (here any point in the Tyrrhenian Sea), and this after a given advection time scale^53^. Then, we inferred from the Lagrangian PDFs the physical connectivity matrix which gives the probability that a particle leaving a given sampling site reaches another sampling site. The combined analysis of LPDFs and connectivity matrix allows for a complete drawing of the seascape and its related transport dynamic.

### Model validation

Our ROMS model has been validated following two different approaches. As a first step, we compared model tracer patterns at the surface to satellite observations in the visible band. More specifically, we visually compared the model sea surface salinity and chlorophyll-a estimates from ocean colour data, as both these parameters are very effective in identifying the river plumes and the surface circulation in the coastal areas. Indeed, the filaments and steep gradients created by eddy stirring and turbulent advection at the (sub)mesoscale very clearly mark the small scale circulation features along the Tyrrhenian coasts. This qualitative comparison was carried out using the 1 km resolution MODIS-Aqua chlorophyll-a daily data distributed by CMEMS. The analysis revealed that the surface current variability, mainly driven by rapid changes in the wind-stress, was generally well reproduced in our simulations. The second approach consisted in comparing model data with the *in situ* observations collected during the EVATYR surveys. This analysis included both the comparison of observed tracer distribution (see Fig. 6) and a quantitative evaluation of the differences between observed salinity (and temperature) and model matchup data. Surface salinity, in particular, showed a significant improvement in the root-mean-square error with respect to that of the MED-MFC model (from 0.6 to 0.4), which is an expected consequence of the inclusion of observed discharges from both major and minor rivers in our ROMS configuration.

### Acoustic data collection and association anchovy distribution and environmental processes

The echosurvey was carried out in the period 17 May–9 June 2013. A zig-zag sampling strategy was adopted in the southern part of the study area due to the very narrow continental shelf characterizing this sector, while in the northern part a parallel transects design was adopted. At each transect vertex, vertical profiles of salinity and temperature were also collected by means of SBE911 plus CTD probe. During the cruise, acoustic data were collected at vessel speed of about 8-10 knots by means of Simrad EK60 scientific echosounder working with a split-beam transducer at 38 kHz; the system was calibrated following standard techniques^54^. Obtained data were post-processed using Myriax Echoview software and adopting an Elementary Distance Sampling Unit (EDSU) of one nautical mile (nmi, 1.852 km). During the survey, midwater pelagic trawl sampling was carried out in order to characterize the observed echoes in terms of species compositions and to investigate the length frequency distribution of collected species. Anchovy density (t/nmi^2^) for each EDSU was evaluated by merging the biological and acoustic data, based on the nearest haul method^55^.

To characterize the environmental processes driving differences in ecotype proportions between the Northern and Southern sectors of the Southern/Central Tyrrhenian Sea, a Principal Component Analysis was carried out. Further, the link between anchovy biomass and environmental drivers was investigated by means of Quotient Index analysis in order to verify if identified processes had some influence on anchovy biomass distribution. To this aim, the environmental parameters considered were (i) salinity and temperature at 5 meters depth (derived by CTD vertical profiles collected during the survey); (ii) satellite derived Chl-a values (Chl-a_sat_); (iii) distance from the nearest river mouth and (iv) depth.

Since the spatial resolution of CTD sampling and Chl-a_sat_ product were lower than the one of acoustic dataset, salinity and temperature values were interpolated over 1NM grid by means of kriging, while the Chl-a_sat_ was resampled at 1NM resolution by means of bilinear spline interpolation. Interpolation and resampling were performed by means of GRASS GIS software.

Principal Component Analysis (PCA) was carried out separately in each sector to better discriminate environmental drivers acting in the two areas. The association between anchovy biomass and identified environmental processes was investigated by means of Quotient Index analysis^34, 56^ (QI). In this study the PCA scores values were used as environmental factor since they represent identified processes as linear combination of all or some of the original environmental variables. The significance of association/avoidance was tested using randomization procedure^57^. In order to avoid the presence of few observations in the histogram tails (strongly affetting the resampling procedure), the histogram intervals were computed by balancing the number of observations in each bin.

### Trophic analyses

A portion of white muscle was extracted from each fish individual, oven-dried (60 °C for 24 h), powdered and weighted (0.5 mg) into tin capsules for isotopic analysis. The δ^13^C values of fishes were no normalized for lipid concentration, being the C:N ratio of anchovies <3.5^58^. Stable isotope measurements were carried out by ThermoFisher Flash EA 1112 elemental analyzer coupled to a Thermo Electron Delta Plus XP isotope ratio mass spectrometer (IRMS). Samples were run against blank cups and known urea standards of certificated isotopic composition. Three capsules of urea were analysed at the beginning of each sequence and one every six samples as a quality control measure. Experimental precision (based on the standard deviation of replicates of the internal standard) was < 0.3‰ for δ^15^N and < 0.4‰ for δ^13^C. The δ^15^N and δ^13^C values were obtained in parts per thousand (‰) relative to Vienna Pee Dee Belemnite (VPDB) and atmospheric N_2_ standards respectively, according to the following formula:

$${\delta }^{13}{\rm{C}}\,{\rm{or}}\,{{\rm{\delta }}}^{15}{\rm{N}}=[({{\rm{R}}}_{{\rm{sample}}}{/R}_{{\rm{standard}}})-1]\times {10}^{3},\,{\rm{where}}\,{\rm{R}}={}^{13}{\rm{C}}/{}^{12}{\rm{C}}\,{\rm{or}}\,{}^{15}{\rm{N}}/{}^{14}{\rm{N}}.$$

### Anchovy diet

In order to describe the diet composition, 60 anchovies were selected from 6 sampling areas (Table 1), with preference for the same individuals used for the SIA. Stomachs were preserved individually in a buffered 4% formaldehyde-seawater solution. The stomach contents of ten anchovies from the same sampling site were pooled and diluted in a known volume of filtered seawater. Subsamples of this volume were observed under the stereo-microscope using 40 to 160 magnification. Data were expressed as number of prey per anchovy and organized in a matrix of prey items vs sampling ara. A cluster analysis based on similarity in feeding habits was applied using the Primer v.6 package^59^. The Bray-Curtis coefficient of similarity^60^ and the complete linkage were applied to square root transformed data.

## Electronic supplementary material

Supplementary Informations
