# Supplementary material for: Insights on the drivers of genetic divergence in the European anchovy
Source: Sci Rep. 2017 Jun 23;7:4180. doi: 10.1038/s41598-017-03926-z (PMC5482869; doi:10.1038/s41598-017-03926-z)
Supplement: Supplementary file 1 — Supplementary Informations [file 41598_2017_3926_MOESM1_ESM.pdf]

SUPPLEMENTARY INFORMATIONS:

Insights on the drivers of genetic divergence in the European anchovy

Gaetano Catanese[1,A,*], Romain Watteaux[1,*], Iratxe Montes[2], Marco Barra[3], Paola Rumolo[3], Diego Borme[4], Bruno Buongiorno Nardelli[3], Vincenzo Botte[3], Maria Grazia Mazzocchi[1], Simona Genovese[3], Iole Di Capua[1], Mikel Iriondo[2], Andone Estonba[2], Paolo Ruggeri[5,6], Valentina Tirelli[4], Vincenzo Caputo-Barucchi[5], Gualtiero Basilone[3], Angelo Bonanno[3], Daniele Iudicone[1] & Gabriele Procaccini[1]

[1]Department of Integrative Marine Ecology, Stazione Zoologica Anton Dohrn-SZN, Villa Comunale, 80121 Napoli, Italy;

[2]Department of Genetics, Physical Anthropology and Animal Physiology, University of the Basque Country-UPV/EHU, Sarriena auzoa z/g, Leioa - Bizkaia, Spain;

[3]Institute for Coastal and Marine Environment (IAMC), Consiglio Nazionale delle Ricerche-CNR, DetachedUnits of Capo Granitola (TP) and Naples, Italy;

[4]Istituto Nazionale di Oceanografia e di Geofisica Sperimentale-OGS, Department of Oceanography, Via A. Piccard 54, 34151 Trieste, Italy;

[5]Dipartimento di Scienze della Vita e dell'Ambiente-DiSVA, Università Politecnica delle Marche, via Brecce Bianche, 60131 Ancona - Italy;

[6]University of Miami, RSMAS, 4600 Rickenbacker Causeway, 33149, Miami, Florida (USA).

Figure S1 STRUCTURE ANALYSIS. The number of sub-populations (ΔK) was determined using the method of Evanno[39] based on the rate change in the log probability of data between successive K values, and implemented in Structure Harvester. ΔK reached its maximum value when K = 2

a) Proportional membership (Q) of each accession at K = 2. Atlantic sampling sites considered as coastal in Montes *et al.* [20] are labelled with red letters. b) Plot of ΔK.

a)

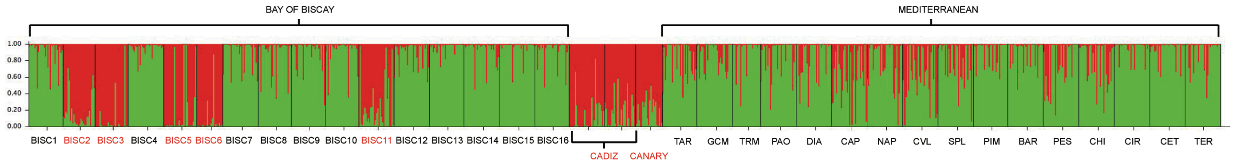

b)

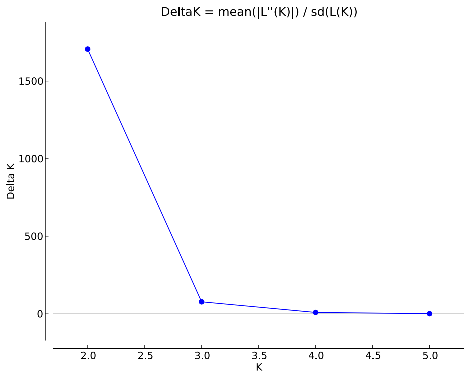

K	Reps	Mean LnP(K)	Stdev LnP(K)	Ln'(K)	|Ln''(K)|	Delta K
1	10	-108190.500000	0.081650	-	-	-
2	10	-98576.225000	4.594471	9614.275000	7839.500000	1706.289900
3	10	-96801.450000	13.433416	1774.775000	1037.975000	77.268135
4	10	-96064.650000	7.079313	736.800000	62.325000	8.803821
5	10	-95390.175000	616.9046025	674.475000	552.100000	0.894952
6	10	-94163.600000	107.309583	1226.575000	-	-

Figure S2 Genetic barriers predicted by BARRIER (version 2.2), employing Monmonier's maximum difference algorithm to highlight geographical features corresponding to pronounced genetic discontinuity in Tyrrhenian Sea (red bold lines with arrows). Sampling sites (red points) are shown.

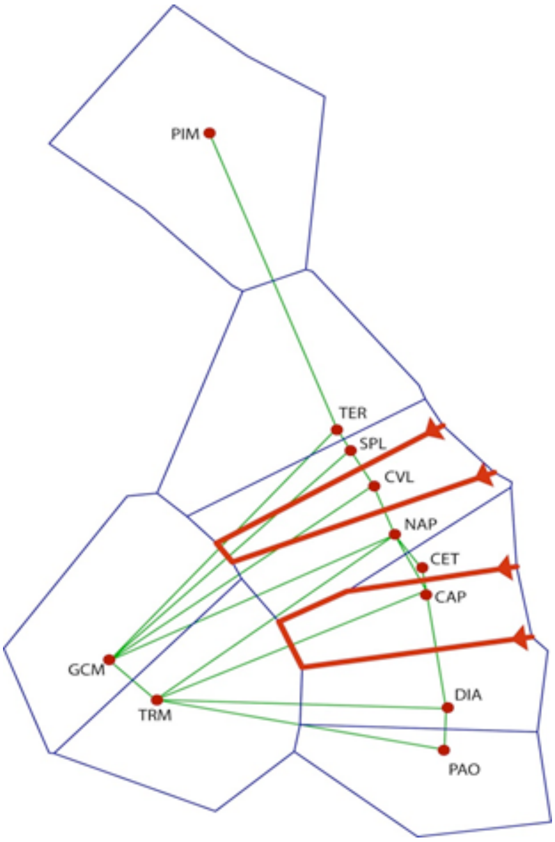

Figure S3 Detection of outlier SNPs.a) BayeScan Log 10(q value) are shown on the x-axis and F_{ST}-values on the y axis. Log10(q-value): decision factor in logarithmic scale (base 10) to determine selection; b) Lositan graphical output with the simulated confidence area for neutral loci (grey color band) with loci from the original empirical dataset represented as dots.Candidate positive selection outliers are situated in the red band.

a)

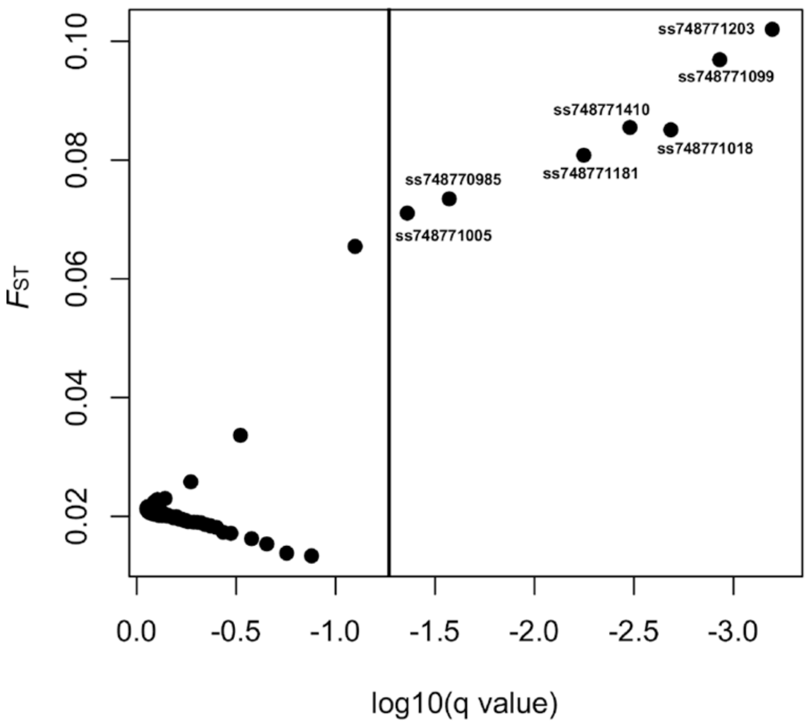

b)

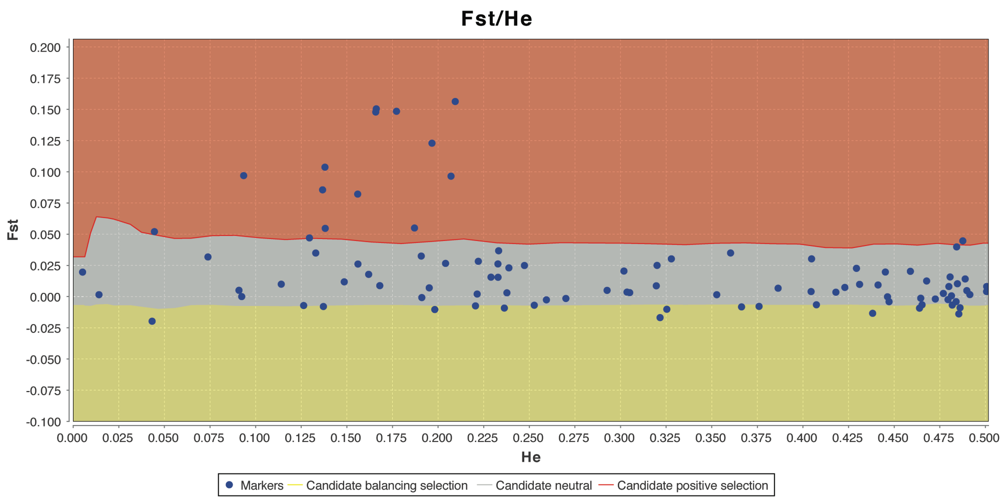

Figure S4 PCoA of Mediterranean and Atlantic samples with (a) only putative neutral markers (variance of axis 1: 65.11%; variance of axis 2: 13.27%); and (b) with putative outliers markers (variance of axis 1: 98.58%; variance of axis 2: 0.85%). Atlantic coastal populations are labelled with red letters. PCoA of the Mediterranean and Atlantic samples grouped by sampling locality with (c) only putative neutral markers (variance of axis 1: 63.2%; variance of axis 2: 18%); and (d) with putative outliers markers (variance of axis 1: 94.9%; variance of axis 2: 3.8%). In circles we highlighted the different ecotypes. PCoA of Tyrrhenian adult anchovies and eggs sample sites (e) with putative neutral markers (variance of axis 1: 24.41%; variance of axis 2: 9.20%) and (f) putative outlier markers (variance of axis 1:84.5%; variance of axis 2: 8.44%).

a)

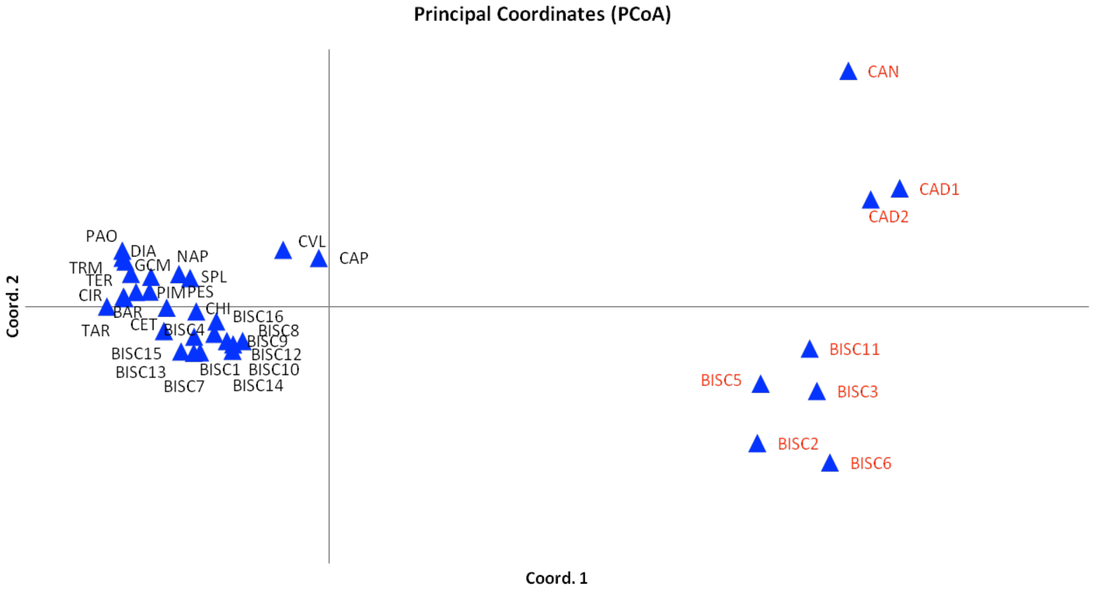

b)

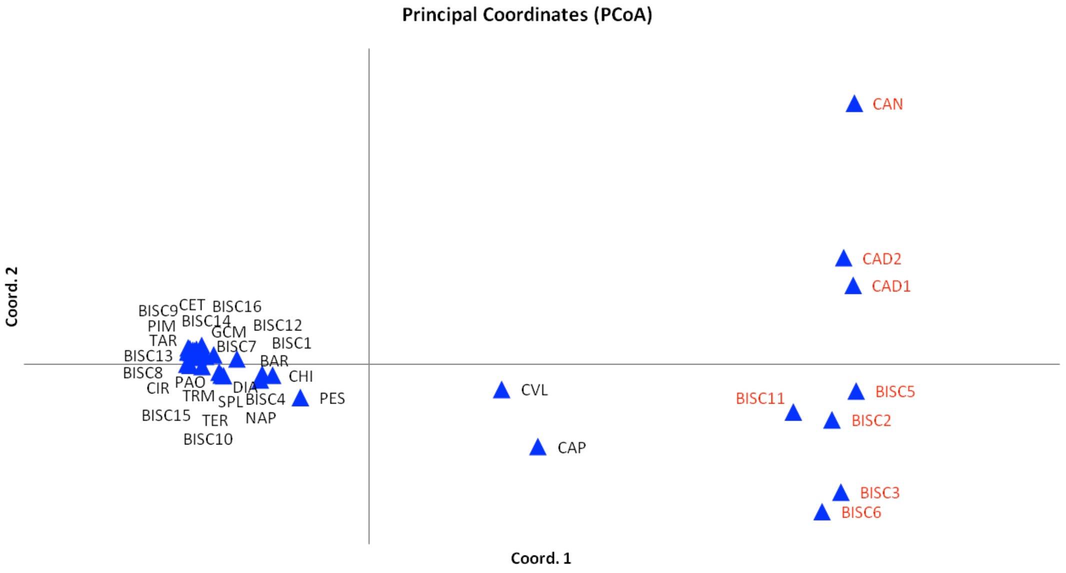

c)

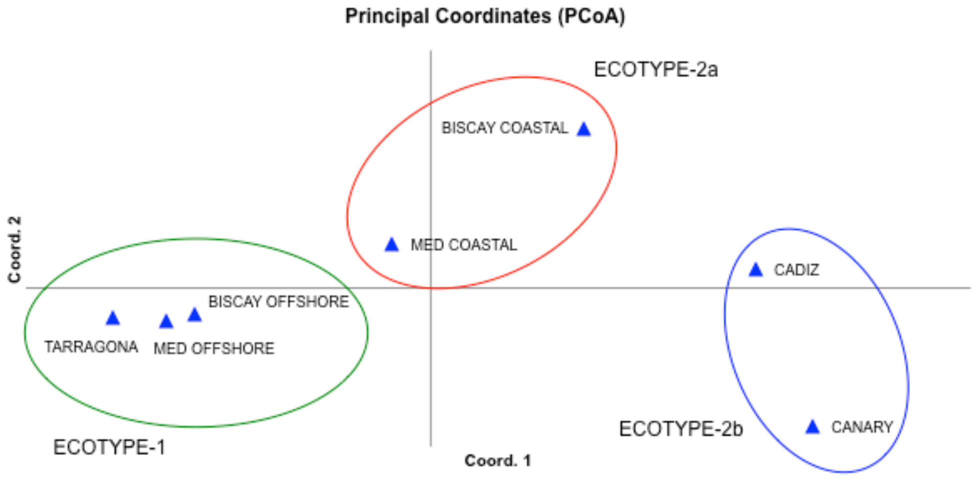
Principal Coordinates (PCoA)
ECOTYPE-2a
BISCAY COASTAL
MED COASTAL
CADIZ
BISCAY OFFSHORE
TARRAGONA
MED OFFSHORE
ECOTYPE-2b
CANARY
ECOTYPE-1
Coord. 1
Coord. 2

d)

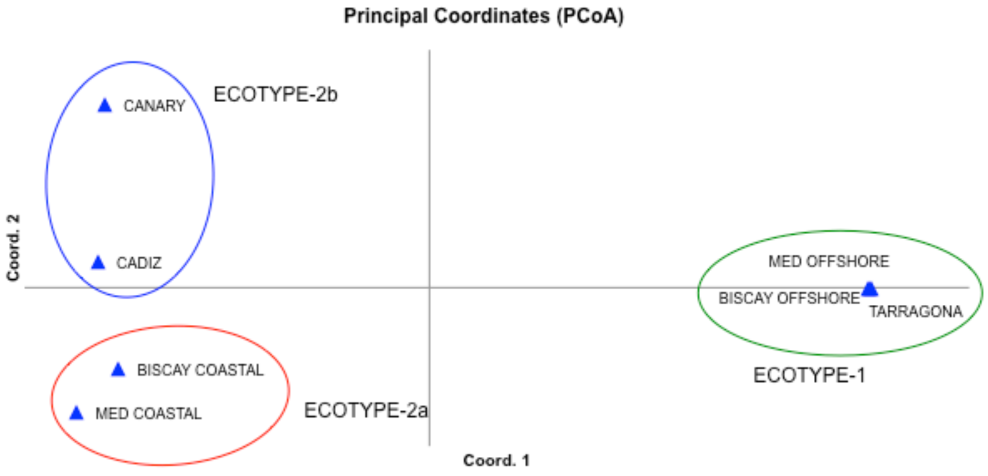
Principal Coordinates (PCoA)
CANARY
ECOTYPE-2b
CADIZ
MED OFFSHORE
BISCAY OFFSHORE
TARRAGONA
ECOTYPE-1
BISCAY COASTAL
MED COASTAL
ECOTYPE-2a
Coord. 1
Coord. 2

e)

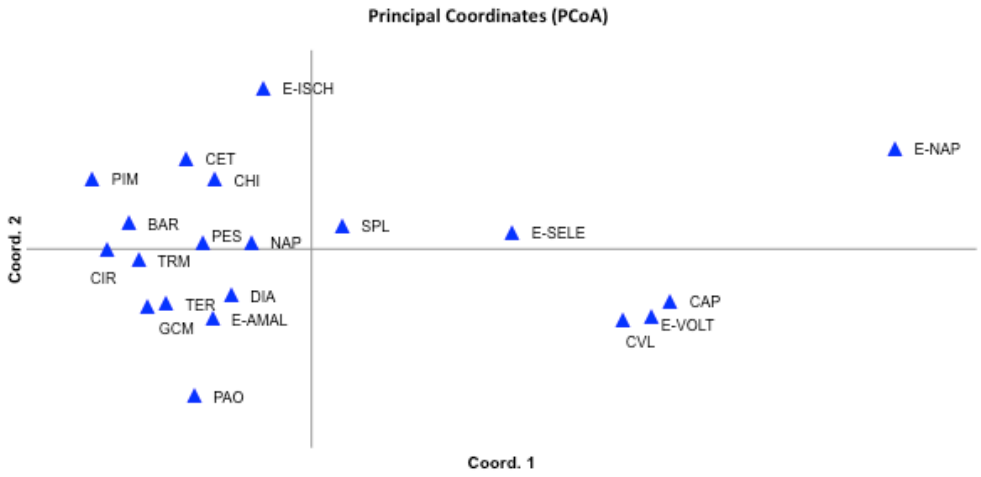
Principal Coordinates (PCoA)
E-ISCH
E-NAP
CET
PIM
CHI
BAR
PES
NAP
SPL
E-SELE
Coord. 2
CIR
TRM
DIA
CAP
TER
E-AMAL
E-VOLT
GCM
CVL
PAO
Coord. 1

f)

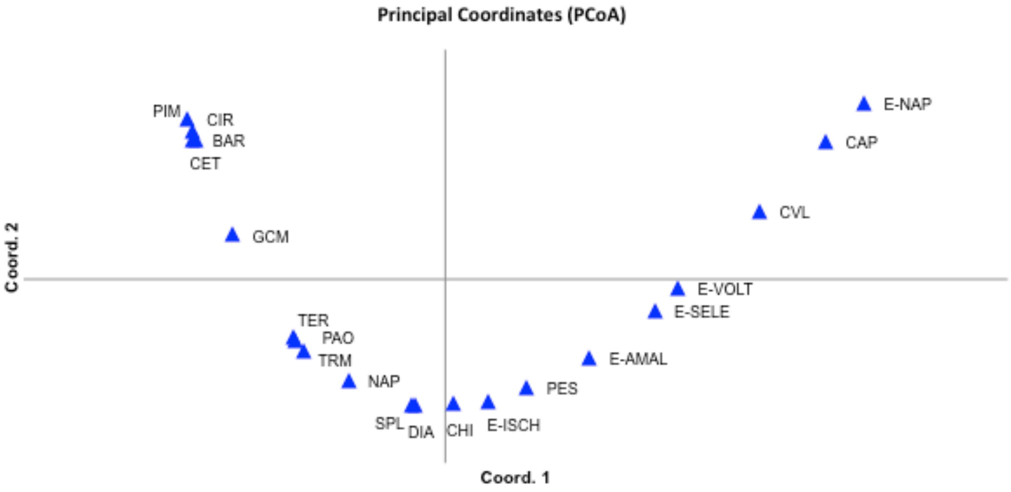
Principal Coordinates (PCoA)
E-NAP
PIM
CIR
BAR
CAP
CET
CVL
GCM
Coord. 2
E-VOLT
E-SELE
TER
PAO
TRM
E-AMAL
NAP
PES
SPL
DIA
CHI
E-ISCH
Coord. 1

Figure S5. Characterization of the zooplankton community in a sub-set of stations in which anchovies were collected; a) total zooplankton biomass; b) relative abundance of copepods; c) abundance of most representative groups

a)

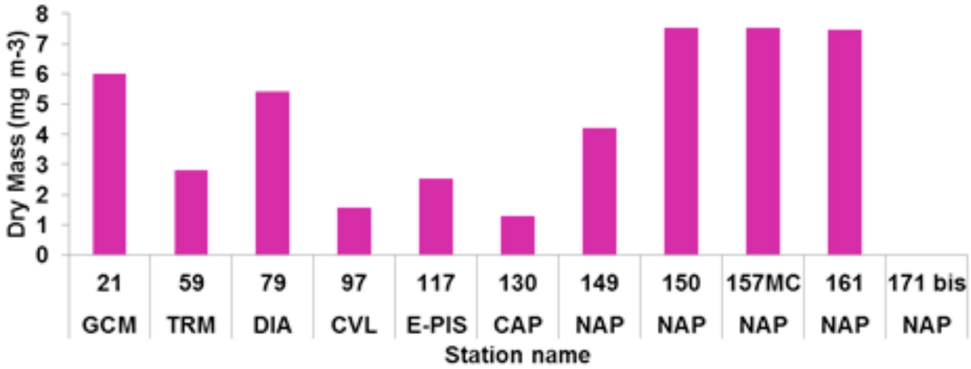

b)

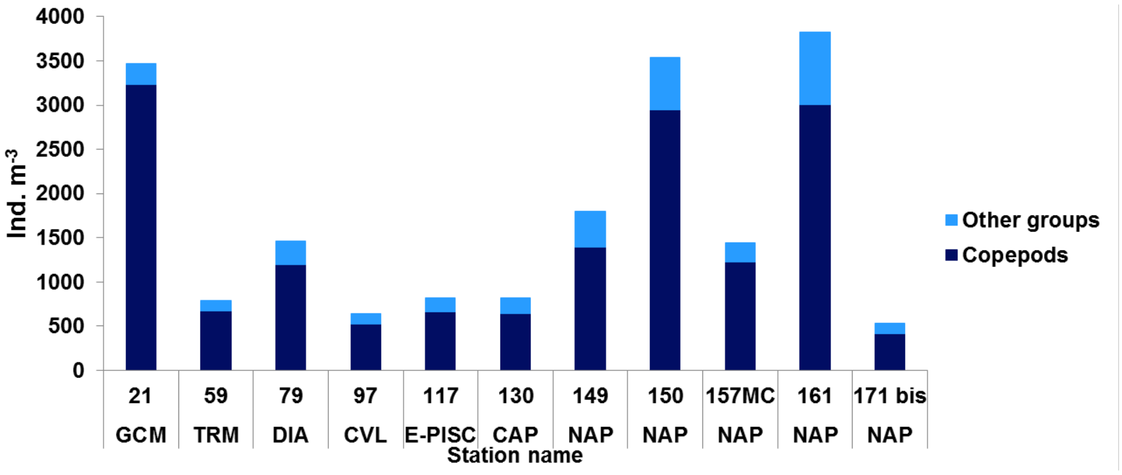

c)

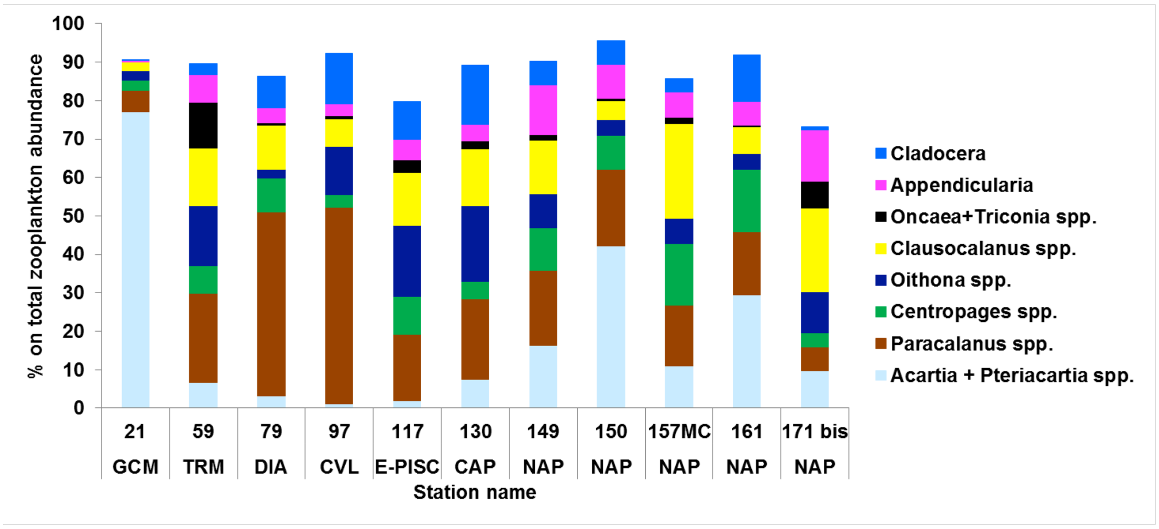

Figure S6 (a) Clustering of the different areas obtained using the diet of anchovy as descriptor, (b) MDS representation of the similarity among areas obtained using the diet of anchovy as descriptor.

a)

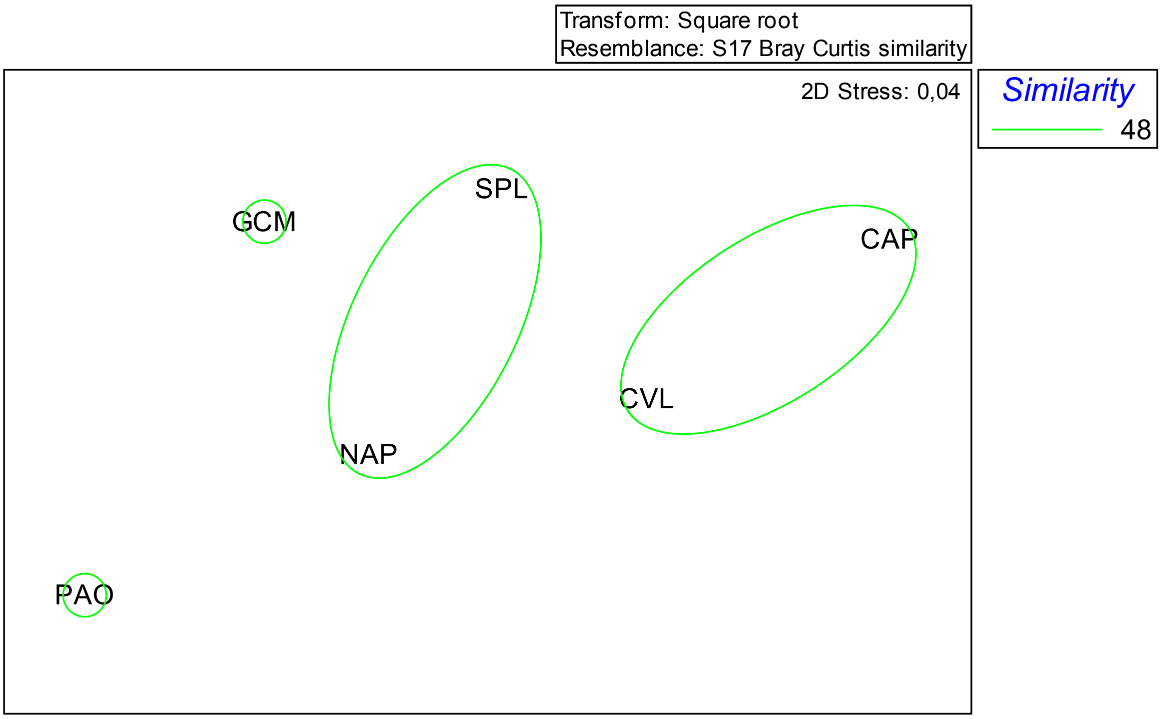

b)

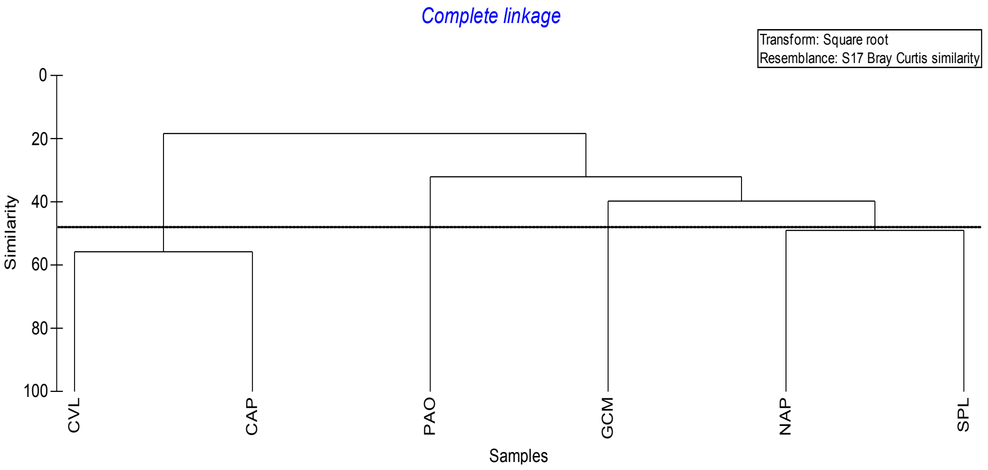

Figure S7: Boxplot of $\delta^{15}N$ (top panel) and $\delta^{13}C$ (bottom panel) values recorded in each considered station.

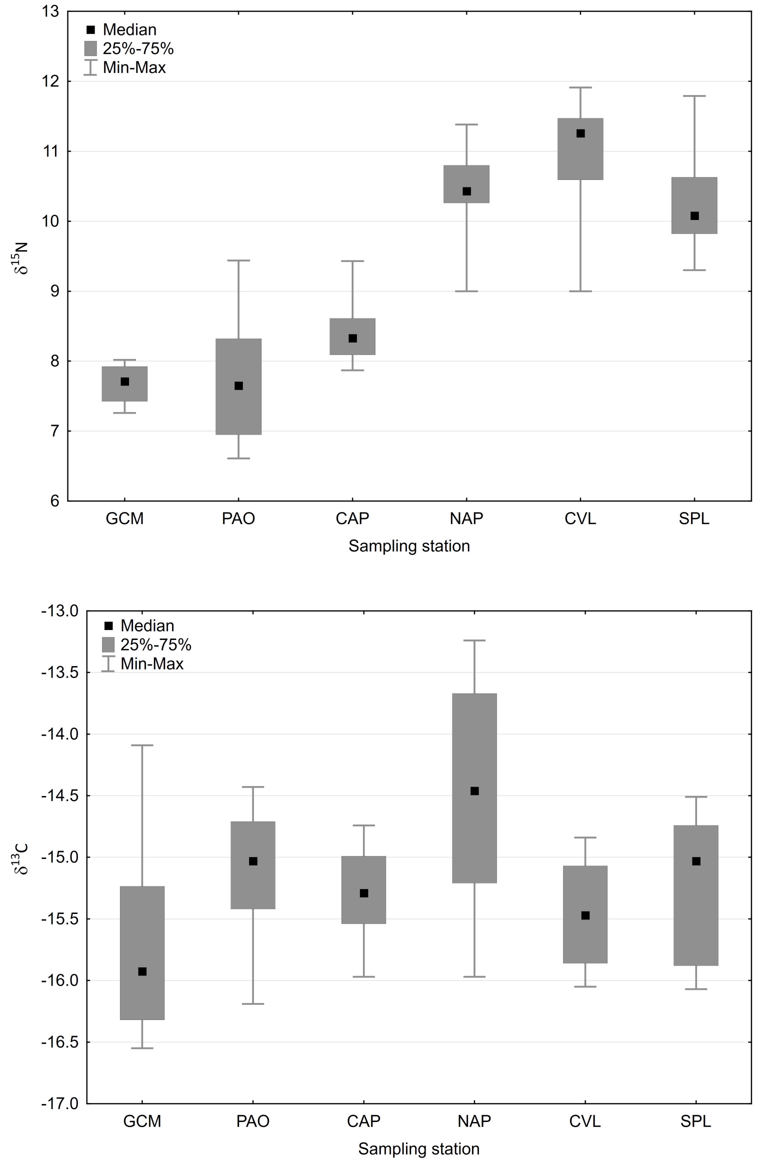

Table.S1 Anchovy diet composition expressed by percentage.

Group	Prey item	SPL	CVL	NAP	CAP	PAO	GCM
BACILLARIOPHYCEAE	*Coscinodiscus* spp.	0.0	0.8	0.0	0.8	0.0	0.0
DINOPHYCEAE	*Dinophysis fortii*	0.0	0.0	0.6	0.0	0.0	0.0
	Phalacroma rotundatum	0.0	0.0	0.0	0.0	0.4	0.0
MOLLUSCA	Atlantidae indet.	0.0	0.4	1.8	5.5	2.8	0.0
	Bivalvia veliger	0.0	3.1	0.0	1.6	0.0	0.0
POLYCHAETA	Polychaeta larvae	0.0	0.4	0.0	0.0	0.0	2.3
CLADOCERA	*Podon intermedius*	0.0	0.0	0.0	0.8	0.4	0.0
OSTRACODA	Ostracoda indet.	0.0	0.0	0.0	0.0	6.8	0.0
Calanoida	*Acartia clausi*	0.0	0.4	0.0	0.0	0.0	0.0
	Acartia spp.	0.0	1.5	0.0	0.8	0.0	0.0
	Calanus helgolandicus	0.0	0.0	0.0	0.0	0.4	0.0
	Candacia giesbrechti	1.3	0.0	8.6	0.0	0.0	0.0
	Candacia spp.	6.7	0.8	12.3	0.0	1.1	2.3
	Centropages typicus	13.4	3.8	16.6	0.8	1.8	2.3
	Centropages spp.	0.0	3.5	5.5	0.8	0.0	0.0
	Clausocalanus sp.	0.0	1.5	1.8	0.0	1.1	0.0
	Euchaeta sp.	0.0	0.0	0.0	0.0	0.4	0.0
	Heterorhabdus spp.	0.0	0.0	0.0	0.0	0.7	0.0
	Isias clavipes	0.0	0.0	0.0	0.0	0.4	0.0
	Lucicutia spp.	0.0	0.0	1.8	0.0	0.0	0.0
	Paracalanus spp.	0.0	1.5	1.2	1.6	0.0	0.0
	Pleuromamma abdominalis	0.0	0.0	0.6	0.0	0.4	0.0
	Pleuromamma gracilis	0.0	0.0	0.0	0.0	0.4	0.0
	Pleuromamma sp.	0.0	0.4	0.6	0.0	0.4	0.0
	Temora stylifera	5.3	0.0	2.5	0.0	0.0	0.0
	Calanoida prosoma<500	0.0	1.9	0.0	0.0	0.0	0.0
	Calanoida 500<prosoma<1000	0.0	9.6	3.1	15.6	0.4	6.9
	Calanoida 1000<prosoma<1500	2.7	6.2	6.8	0.8	2.1	18.4
	Calanoida 1500<prosoma<2000	1.3	1.2	0.6	0.0	1.4	0.0
	Calanoida prosoma>2000	0.0	0.0	3.1	0.0	0.0	0.0
Cyclopoida	*Oithona* cf. *nana*	0.0	1.5	0.0	0.0	0.0	0.0
	Oithona cf. *plumifera*	1.3	1.9	0.6	0.8	0.0	0.0
	Oithona setigera	0.0	0.0	0.0	0.0	0.7	0.0
	Oithona cf. *similis*	0.0	0.4	0.0	0.8	0.0	0.0
	Oithona spp.	0.0	0.8	0.0	0.0	0.0	0.0
Poecilostomatoida	*Corycaeus typicus*	1.3	2.3	0.0	0.0	0.0	0.0
	Corycaeus spp.	5.3	0.4	0.0	0.0	0.7	0.0
	Farranula sp.	1.3	5.0	0.6	25.8	0.0	0.0
	Urocorycaeus sp.	0.0	0.0	0.6	0.0	0.0	0.0
	Vettoria sp.	0.0	0.0	0.0	0.0	0.4	2.3
	Corycaeidae indet.	2.7	0.0	1.2	0.0	0.0	0.0
	Oncaea venusta	0.0	0.0	0.0	0.0	0.4	0.0

	Oncaea prosoma<350	6.7	2.7	1.2	7.0	1.4	20.7
	Oncaea 350<prosoma<600	20.0	5.8	4.9	5.5	27.7	25.3
	Oncaea prosoma>650	1.3	0.0	0.6	0.0	3.9	2.3
	Triconia conifera	0.0	0.0	2.5	0.0	12.4	2.3
Harpacticoida	*Euterpina acutifrons*	4.0	31.9	0.0	25.8	0.0	0.0
	Microsetella spp.	14.7	4.6	1.8	0.0	2.5	11.5
	Harpacticoida indet.	0.0	0.0	0.0	0.0	0.4	0.0
	Copepoda nauplius	0.0	0.4	0.0	1.6	0.0	0.0
	Copepoda (not Calanoida)	0.0	0.4	0.0	0.0	0.0	0.0
AMPHIPODA	Caprellidae indet.	0.0	0.0	0.0	0.0	0.4	0.0
DECAPODA	Decapoda zoea	1.3	0.0	1.2	0.8	0.0	0.0
	Decapoda mysis	0.0	0.0	0.6	0.0	0.0	0.0
	Decapoda megalopa	0.0	0.0	0.6	0.0	0.0	0.0
	Decapoda indet.	0.0	0.0	8.3	0.0	0.2	3.4
APPENDICULARIA	*Oikopleura* spp.	0.0	0.4	2.5	0.0	26.7	0.0
	Appendicularia indet.	0.0	0.4	0.0	2.3	0.0	0.0
CHAETOGNATHA	Sagittoidea indet.	2.5	0.3	3.3	0.1	0.6	0.1
TELEOSTEA	Teleostea larvae indet.	0.0	0.0	0.0	0.0	0.4	0.0
others	Invertebrata eggs spheric	2.7	1.5	0.0	0.8	0.0	0.0
	Invertebrata eggs elliptical	2.7	1.2	0.6	0.0	0.0	0.0
	Invertebrata eggs irregular	0.0	0.0	0.6	0.0	0.0	0.0
	Pollen grains	0.0	0.8	0.6	0.0	0.4	0.0
	Carabidae indet.	1.3	0.0	0.0	0.0	0.0	0.0
	Insecta indet.	0.0	0.4	0.0	0.0	0.0	0.0
